# Monocyte Chemokines Enhance Atherosclerotic Plaque Necrosis After Bacterial Kidney Infection

**DOI:** 10.1096/fj.202505052R

**Published:** 2026-04-03

**Authors:** Lena Possenriede, Peyman Falahat, Marie Foerster, Georg W. Sendtner, Julia Miranda, Leonie Richard‐Stropahl, Uta Scheidt, Christian Kurts, Florian Wagenlehner, Ekaterina K. Koltsova, Sibylle von Vietinghoff

**Affiliations:** ^1^ Nephrology Section, Medical Clinic 1 University Hospital Bonn, Rheinische Friedrich‐Wilhelms‐Universität Bonn North Rhine‐Westphalia Germany; ^2^ IMMEI University Hospital Bonn, Rheinische Friedrich‐Wilhelms‐Universität Bonn North Rhine‐Westphalia Germany; ^3^ Clinic for Urology, Pediatric Urology, and Andrology Justus‐Liebig‐Universität Gießen Gießen Hesse Germany; ^4^ Department of Medicine and Biomedical Sciences Cedars‐Sinai Health Science University Los Angeles California USA

## Abstract

Cardiovascular event rate increases after acute infections, including urinary tract infections (UTI). Vascular inflammation is a major contributor to atherogenesis and plaque instability. The mechanistic pathway by which UTI impacts atherosclerosis has not been determined. Cardiovascular events were analyzed in a patient cohort, and atherosclerosis development was assessed in *Ldlr* ^*−/−*^ mice after a pyelonephritis (PN) episode. Inflammatory gene expression profiles of murine and human atherosclerotic vessels were analyzed. Monocyte mobilization was studied by *Ccr2* ablation in mice and in human primary monocytes in vitro. UTI and cardiovascular event rates are significantly associated in a propensity‐score matched cohort of kidney graft recipients. Atherosclerotic aortic root plaque necrotic core area significantly increased in mice after PN. Monocyte chemotactic cytokine CCL2 was systemically elevated during healing, and its receptor CCR2 on bone marrow cells was required for increased plaque necrotic core formation, but not kidney host response or healing from PN. CCR2‐mediated monocyte mobilization as demonstrated in mixed bone marrow chimeras. PN upregulated innate immune genes in the aortas during plaque development. Monocyte chemokine CCL8 was upregulated in murine atherosclerotic aorta after PN. Human *CCL8* expression is associated with an inflammatory signature in human atherosclerotic plaques and systemically increased in acute human PN. As potential underlying mechanisms, CCL8 promoted human primary CCR2^+^ monocyte migration and survival in vitro. Monocyte mobilization mediates increased atherosclerotic inflammation and plaque necrosis after bacterial kidney infection. Our data demonstrate the impact of this inflammatory pathway mediating organ interaction for enhanced cardiovascular risk.

## Graphical abstract

Cardiovascular event rates rise after acute infections. After a single pyelonephritis episode in *Ldlr* ^*−/−*^ mice, atherosclerotic plaque necrotic core size increased, mediated by chemokine receptor CCR2‐induced monocyte mobilization from the bone marrow. Aortic gene regulation after PN paralleled human atherosclerotic plaques with an inflammatory signature, including increased human CCR2 ligand CCL8. CCL8 induced human monocyte chemotaxis and inhibited cell death in vitro. Inflammatory monocyte chemotaxis may be considered as a mechanistic target to prevent plaque destabilization after bacterial kidney infection.

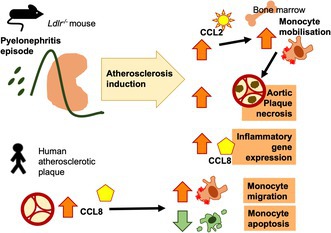

## 1 Introduction

Cardiovascular events continue to range among the most common causes of death worldwide [1]. They are caused by atherosclerosis, a pathological thickening of the arterial vessel wall that predisposes to stenosis and, if ruptured or eroded, acute thrombosis. Risk factors include hyperlipidemia and chronic inflammation. Clinically, this is highlighted by the recent success of emerging anti‐inflammatory strategies [2, 3, 4]. Also, following viral and bacterial infections that promote systemic inflammation, observational studies have repeatedly detected elevated atherosclerotic event rates [2, 5, 6, 7, 8, 9, 10]. The kidney and urinary system are among the most commonly infected organs, together with the respiratory system. Urinary tract infections (UTI) incidence currently ranges around 400 million per year worldwide [11].

Antibacterial host responses employ innate immune pathways relevant in atherogenesis [5, 12]. However, in vivo experimental approaches to define atherogenic mechanisms during bacterial infection are diverse, comprising a large and partially contradictory body of earlier studies on chlamydial infections [13, 14, 15, 16] and more recent studies in murine models of atherosclerosis and streptococcal pneumonia [17, 18]. Bacterial pyelonephritis (PN) locally and systemically activates innate immune pathways with known pro‐atherogenic effects [19, 20]. To the best of our knowledge, studies investigating atherogenesis after experimental PN and the definition of underlying mechanisms are not currently available.

Chemotactic cytokines (chemokines) promote atherogenesis as delineated in detail in experimental atherosclerosis models [21]. Chemokine receptor CCR2, which mediates monocyte egress from the bone marrow [22], has been implicated in atherogenesis starting from early specific gene‐deficient mouse models [21, 23]. A proatherogenic function agrees with human data. Here, a rare CCR2 deletion mutant is associated with a lower cardiovascular event rate [24]. However, some of the earlier murine results may have been affected by the genetic background of specific gene‐deficient mouse lines [25], and inhibitor studies yielded mixed results. Newer mechanistic murine studies primarily propose that CCL2‐CCR2 interactions promote early lesions and act cooperatively with toll‐like receptors (TLR), sensors of the bacterial cell wall constituent lipopolysaccharide [21, 26, 27]. Emerging murine data also suggest a pro‐atherogenic role for elevated or dynamic levels of its ligand CCL2 (alternate name: MCP‐1), including diurnal variation [28] and a rise after renal parenchymal ischemia–reperfusion injury [29]. Whether CCR2 is involved in altered plaque formation and elevated cardiovascular risk after bacterial infections, namely PN, has not been reported.

We recently established a model of acute and chronic pyelonephritis in atherosclerosis‐prone *Ldlr* ^*−/−*^ mice [30, 31], which we now employed to study the impact of a single PN episode on subsequent atherosclerotic plaque formation. Our data demonstrate a mechanistic role of chemokine receptor CCR2 in the promotion of atherosclerotic necrotic core formation during this process. We continued to define the regulation of CCR2 and its ligands in murine and human atherosclerotic plaques and to investigate its most regulated human ligand, CCL8.

## 2 Methods

### 2.1 Patient Cohorts

Human sample and clinical data analysis were approved by the University Clinic Bonn ethics board, and data use committees (UKB 039/22, DIZ67 and 154, and UK Giessen 280/20), and in agreement with the principles of the Declaration of Helsinki.

For analysis of the association of cardiovascular event rate and upper UTI, electronic patient records of adult renal transplant recipients with at least one outpatient visit post‐transplantation at the University Clinic Bonn transplantation center from January 1st through March 31st, 2022, were analyzed. Kidney transplantations were conducted according to the Declaration of Istanbul. The local ethics board waived informed consent for this retrospective analysis. All patients who were followed for at least 100 days since transplantation were included. Demographic data, clinical diagnoses, and laboratory values, including microbiology findings, were extracted from the records. To control for potential confounders, propensity score matching analysis was performed with the covariates age, gender, diabetes mellitus, body mass index, hypertensive nephropathy, diabetic nephropathy, reflux and infection, cystic kidney diseases, and Alport's syndrome, graft vintage, living donor status, baseline eGFR, and immunosuppressive therapy. Propensity scores were estimated using logistic regression, and 1:1 nearest‐neighbor matching without replacement was applied using a caliper width of 0.2 standard deviations of the logit of the propensity score. 12 patients were excluded due to missing data, and 21 patients remained unmatched. Urinary foreign body or bladder augmentation after transplantation had to be excluded, as all patients were grouped in the “high” category. Group balance was assessed using standardized mean differences and the Kolmogorov–Smirnov statistic before and after matching (Figure S1). Outcomes were analyzed using a linear regression model, and Cohen's d with corresponding 95% confidence intervals was calculated to quantify the effect size. The analysis was conducted using R (version 4.3.1 in RStudio 2025.05.1 + 513) with the *MatchIt* and *cobalt* packages [32].

Plasma from patients with acute pyelonephritis diagnosed at the Urology department, University of Giessen (*n* = 30, 83% females, age 37.1, range 16–86 years) was compared to healthy controls (*n* = 7, 75% females, age 27, range 23–33 years).

### 2.2 Animals, Murine Pyelonephritis Model, Induction of Atherosclerosis, and Bone Marrow Transplantation

Wildtype (wt, CD45.1), LDL receptor‐deficient (*Ldlr* ^*−/−*^), and *Ccr2* ^*−/−*^ mice (both CD45.2, all on C57Bl/6 background, Jackson Labs, Bar Harbor, ME) were genotyped by PCR and kept in specific‐pathogen‐free conditions. Animal experiments were approved by Landesamt für Natur‐, Umwelt‐ und Verbraucherschutz, North Rhine‐Westphalia, Germany, in accordance with the guidelines from Directive 2010/63/EU of the European Parliament on the protection of animals used for scientific purposes (#81–02.04.2022.A125, #81–02.04.2023.A395).

For induction of pyelonephritis, female *Ldlr* ^*−/−*^ mice were anesthetized with isoflurane, and uropathogenic *Escherichia coli* strain 536 was instilled into the bladder twice at a 3‐h interval as described [30, 33]. Bacterial clearance was defined as the absence of bacterial colony‐forming units (CFU) in the urine within four weeks of infection and the kidneys at sacrifice, as well as the absence of macroscopic or microscopic evidence of abscess formation [30]. One week after infection, mice were put on a high‐fat diet (Harlan Teklad 88 137, Sniff) for the indicated time periods. Bone‐marrow transplantation was performed with unfractionated bone marrow after lethal irradiation in a MultiRad225 irradiation system (Precision, Madison, CT). Euthanasia was by cervical dislocation.

Serum urea and creatinine were measured at the central laboratory of BonnUniversity Hospital. Serum cholesterol and triglycerides were assessed in a Cobas b101 system (Roche, Basel, Switzerland). Full blood counts were analyzed with an automated hematology analyzer (VetABC; ScilVet, Viernheim, Germany).

### 2.3 Histology

Atherosclerotic lesion size was assessed in frozen 5 μm sections obtained in 50 μm intervals covering 500 μm, stained with Oil Red O as described [29]. Necrotic cores were assessed in the sections with the largest lesions. Cap thickness was assessed by averaging ten measurements per aortic root section in up to three consecutive serial sections in each mouse in the sections with the largest lesions. Immunostaining for F4/80 (CI: A3‐1, Bio‐Rad Laboratories, Hercules, CA) and CD3epsilon (Thermofisher Scientific, Waltham, MA) with secondary Alexa Fluor488 donkey anti‐rat and Alexa Fluor555 goat anti‐rabbit (both: Abcam, Cambridge, UK) and DAPI (Dianova, Geneva, Switzerland) was as described [31]. Imaging as a tile scan (20×magnification) using a Leica Stellaris 8 confocal microscope (Leica Biosystems, Wetzlar, Germany) was employed to quantify total nuclei (DAPI), CD3^+^ positive T cells, and F4/80^+^ positive macrophage area within the plaque using QuPath (version 0.5.1; https://qupath.github.io/). Renal tissues were fixed in 4% neutral‐buffered formaldehyde and embedded in paraffin. Periodic acid‐Schiff (PAS) stainings with hemalaun counterstain (Roth) and Masson Trichrome staining of deparaffinized tissues were mounted with Roti Histokitt (all: Roth Werke GmbH, Dautphetal, Germany). Slides were imaged at 20× magnification with an Aperio CS2 slide scanner (Leica Biosystems, Wetzlar, Germany) and analyzed using GIMP (version 2.10.32) for the aortic roots and QuPath (version 0.5.1) for the kidneys.

### 2.4 Cell Culture, Migration, Apoptosis, and OxLDL Uptake Assays and Macrophage Differentiation

Leukocytes were recovered from anonymized buffy coats obtained as a waste product from the blood donor service after local ethics board approval (UKB 039/22). Mononuclear cells were enriched by density gradient centrifugation with Pancoll 1.077 (PAN‐Biotech, Aidenbach, Germany). For assessment of migration, fresh full RPMI with 5*10^5^ cells was added to the upper well of a transwell insert (5 μm, Corning). CCL2 and CCL8 (PeproTech, ThermoFisher Scientific, Darmstadt, Germany) were added to the lower chamber in the indicated concentrations. Migration was quantified by flow cytometry after 1 h essentially as described [34]. 1,1‐dioctadecyl‐3,3,3,3‐tetramethylindocarbocyanine perchlorate (DiI) labeled‐oxLDL (ThermoFisher Scientific) was added at a concentration of 10 μg/mL, and uptake was measured after 3 h [35]. Apoptosis was assessed after 20 h in serum‐free RPMI [33], essentially as described. Adhesion‐enriched human primary macrophages were cultured for seven days with the indicated cytokine concentrations or 0.1% BSA‐solvent control.

### 2.5 Enzymatic Digestion of Tissues and Flow Cytometry

Preparation and enzymatic digestion of kidney, spleen, and aortic tissues were as described [29]. The following antibodies were used: Anti‐mouse: Anti‐CD11b‐PE/BV510 (M1/70), anti‐CD11c‐PE‐Cy7 (N418), anti‐CD45.1‐PE‐Cy7/APC/B421 (A20), anti‐CD45.2‐BV421/APC (104), anti‐CD45‐BV421 (30‐F11) (104), anti‐CD115‐PE/APC (AFS98), anti‐GR1‐APC/PerCp‐Cy5/PE‐Cy7 (RB6‐8C5), anti‐CCR2‐FITC (SA203G11), anti‐F480‐APC (BM8), anti‐Ly6C‐PerCP (HK1.4), anti‐Ly6G‐BV510 (1A8), anti‐MHCII‐FITC (M5/114.15.2), anti‐human: Anti‐CCR2 PE‐(K036C2), anti‐CD11b‐BV510 (ICRF44), anti‐CD14‐FITC or PerCP (HCD14), anti‐CD16‐PerCP or APC (3G8), anti‐CD64‐Pacific Blue (10.1), anti‐CD86‐PE‐Cy7 (BU63), anti‐CD163‐FITC (GHI/61), anti‐CD206‐APC (15‐2), anti‐HLA‐DR‐PE (LN3) (Biolegend, San Diego, CA). Near‐infrared LIVE/DEAD Fixable Dead Cell Stain Kit (Invitrogen, Carlsbad, CA), BD Cytometric Bead Array Mouse Inflammation Kit (both: BD Bioscience, Franklin Lakes, NJ), AnnexinV‐FITC, and LEGENDplex Mouse Inflammation Panel (both: Biolegend, San Diego, CA), CCL8 DuoSet ELISA (R&D Systems) were used according to manufacturers' instructions. Cell numbers were calculated using BD Calibrite APC Beads. Flow cytometry analysis was performed on a Becton‐Dickinson FACSCanto II (BD Bioscience, Franklin Lakes, NJ). Data were analyzed using FlowJo software (Tree Star Inc., Ashland, OR).

### 2.6 RNA Isolation, qPCR, and RNA Sequencing

RNA was isolated using the NucleoSpin RNA Kit (Macherey‐Nagel, Düren, Germany). Yield and purity were determined with a NanoDrop 1000 Spectrophotometer (Thermo Scientific, Waltham, MA). After reverse transcription, real‐time PCR was performed on a LightCycler96 (Roche, Basel, Switzerland) using SYBR‐Green (Biozym Scientific GmbH, Hessisch‐Oldendorf, Germany). Products were confirmed by melting curve analysis and gel electrophoresis. Data were analyzed with HPRT as a reference gene using LinRegPCR software. Primers were as follows (5′–3′): *Hprt*: FP: CAGTCCCAGCGTCGTGATTA, RP: AGCAAGTCTTTCAGTCCTGTC, *Ccr2*: FP: ATCCACGGCATACTATCAACATC, RP: TCGTAGTCATACGGTGTGGTG.

RNA samples were sequenced at the NGS Core Facility in Bonn. 3′ mRNA‐seq was performed using the Lexogen QuantSeq 3′ mRNA FWD Library Prep Kit with 1 000 ng total RNA as input. Sequencing was carried out on an Illumina NovaSeq 6000 platform at a depth of 10 million reads per sample. Adapter trimming was conducted with TrimGalore, and reads were aligned to the mouse reference genome (GRCm38) using the STAR aligner (version 2.7.11b). Quantification was performed with Salmon (version 1.10.3) in alignment‐based mode. Downstream analyses were conducted in R using the Bioconductor package DESeq2 to identify differentially expressed genes between the two groups. Human atherosclerotic plaque gene expression array data from macroscopically unaffected and atherosclerotic areas of 32 patients were accessed at NCBI (GSE43292). For genes represented by multiple probes, only the probe with the highest variance across samples was retained using the matrixStats package (version 1.5.0). Differential gene expression analysis was performed on normalized data using the limma package (version 3.66.0). Gene set enrichment analysis was conducted using t‐statistics for microarray data and a ranking metric defined as log2FoldChange* − log10(padj) for RNA‐seq data with the fgsea package (version 1.36.0) using 1 000 permutations applied using t‐statistics as input. The GO Terms for biological processes (C5, BP) from the Molecular Signatures Database (MSigDB) were accessed through the msigdbr package (version 25.1.1). For comparison of expression profiles of murine kidneys and aortas from the experimental groups and human aortic plaques with *CCL8* expression above and below the median, normalized enrichment scores (NES) were calculated. Regulated gene interactions were visualized using the SwissProt STRING database at https://string‐db.org, showing interactions from text mining, experiments, and database extractions of connected genes [36].

### 2.7 Statistical Analysis

For continuous biological variables, a normal distribution was assumed because most follow a Gaussian distribution. Statistical analysis was conducted using Graphpad Prism. Two‐tailed Student's *t*‐test with Welch's correction in case of unequal variance was used to compare two conditions. If more than two conditions were compared, Sidak's or Dunnett's test was applied after ANOVA as indicated. Data are expressed as mean ± SEM. *P*‐values < 0.05 were considered significant and are indicated: *p < 0.05, **p < 0.01, ***p < 0.001.

## 3 Results

### 3.1 UTI Frequency in Kidney Transplant Recipients Associates With Cardiovascular Event Rate

We investigated the association of urinary tract infections with cardiovascular events in humans in kidney allograft recipients, a high‐risk population for both conditions, with long‐term follow‐up data. Cardiovascular events (myocardial infarction, stroke, revascularization for peripheral vascular disease) were extracted from the records of a prevalent outpatient kidney transplantation recipient cohort (characteristics in Table 1). Expectedly, together with time after transplantation, sex was a major risk factor for UTI (Table 2).

**TABLE 1:** Characterization of the kidney transplant recipient cohort (*n* = 164).

	% (*n*) or mean ± SEM
Basic characteristics	
Age (years)	57.9 ± 1.0
Gender	62% (101) male
Dialysis vintage	2 087 ± 117 days
Previous solid organ transplantation	12% (20)
Living donor	17% (28)
Graft vintage	4 367 ± 267 days
Underlying renal disease	
Diabetic nephropathy	7% (11)
Hypertensive nephropathy	9% (15)
Glomerulonephritis	23% (38)
Cystic kidney diseases and Alport's syndrome	21% (35)
Reflux and infection	13% (21)
Miscellaneous	10% (16)
Unknown	17% (27)
Comorbidities	
Diabetes mellitus	25% (40)
Urinary foreign body or bladder augmentation after Ktx	4.2% (7)
Immunosuppression at first outpatient visit	
Steroids	84% (101)
CNI (Cyclosporine A or Tacrolimus)	100% (119)
Mycophenolate	98% (118)
Graft function at first outpatient visit	
Serum creatinine	2.0 ± 0.1 mg/dL
Serum cystatin C	1.95 ± 0.1 mg/L
eGFR CKD‐EPI (creatinine)	45 ± 2 mL/min
eGFR CKD‐EPI (cystatin C)	43 ± 5 mL/min
Proteinuria (g/g creatinine)	0.16 ± 0.03

**TABLE 2:** Univariate correlations of UTI with clinical risk factors.

Dichotomous variables	
Gender (male = 1)	−0.28 (0.0003)***
Living donor	−0.04 (0.53)
Frequent UTI or reflux before Ktx	−0.08 (0.31)
Foreign body in graft or bladder augmentation	0.20 (0.0085)**
Diabetes mellitus	0.12 (0.13)
Continuous variables	
Age at assessment	0.03 (0.65)
Dialysis vintage	0.076 (0.40)
Time since transplantation	−0.363 (< 0.0001)***
Body mass index (BMI)	−0.016 (0.84)
eGFR CKD‐EPI (creatinine) at first outpatient visit	−0.11 (0.24)
eGFR CKD‐EPI (cystatin C) at first outpatient visit	−0.36 (0.07)

*Note:* Correlations for dichotomous values are given as Spearman's r, and for continuous values as Pearson's r (*p*‐value).

To start to assess a potential excess cardiovascular risk due to UTI, patients were grouped according to UTI rates above and below the median (5.76*10^−4^ infections/day). Propensity score matching was performed for key cardiovascular and UTI risk parameters, as detailed in the methods, resulting in 36 patients per group (love plot inSuppl. Figure S1). UTI rates were 1.4*10^−4^ ± 4.5 × 10^−4^ vs. 29.1*10^−4^ ± 3.2*10^−5^ infections/day. Frequency of cardiovascular events was significantly higher in patients with a high UTI rate (5.1*10^−5^ ± 1.9*10^−5^ vs. 14.7*10^−5^ ± 4.2*10^−5^, *p* = 0.048, Cohen's d = 0.48, 95% CI [0.01–0.94]). Other risk factors did not differ significantly (Table 3).

**TABLE 3:** Characterization of propensity score matched kidney transplant recipients with an infection rate below and above the median (5.7587*10^−4^ /day, *n* = 36/group).

	Low UTI rate	High UTI rate	*p*‐value	SMD
UTI rate (per day)	1.411*10^−4^ ± 4.464 × 10^−4^	2.905*10^−3^ ± 3.155*10^−5^	< 0.0001	
Basic characteristics				
Age (years)	59.64 ± 1.812	58.92 ± 2.062	0.7941	0.0605
Gender (male)	75% (27)	69.4% (25)	0.5742	0.1354
Dialysis vintage	2 167 ± 245.9	2158 ± 229.2	0.9783	0.0059
Previous solid organ transplantation	0% (0)	5.8% (2)	0.1602	0.0555
Living donor	19.4% (7)	27.7% (10)	0.4123	0.2031
Graft vintage (< 365 days)	5.5% (2)	5.5% (2)	> 0.9999	0
Underlying renal disease				
Diabetic nephropathy	8.3% (3)	8.3% (3)	> 0.9999	0
Hypertensive nephropathy	8.3% (3)	11.1% (4)	0.6958	0.1079
Glomerulonephritis	25% (9)	25% (9)	> 0.9999	0
Cystic kidney diseases and Alport's syndrome	27.7 (10)	27.7 (10)	> 0.9999	0
Reflux and infection	8.3% (3)	11.1% (4)	0.6958	0.0946
Miscellaneous	5.5% (2)	8.3% (3)	0.6486	0.0277
Unknown	13.8% (5)	8.3% (3)	0.4604	0.0555
Comorbidities				
Diabetes mellitus	22.2% (8)	25% (9)	0.7851	0.0677
Body Mass Index	25,96 ± 0,5742	25,90 ± 0,6623	0,9381	0.0196
Urinary foreign body or bladder augmentation after Ktx	0% (0)	5.8% (2)	0.1602	0.0555
Immunosuppression at first outpatient visit				
Steroids	83.3% (30)	80.5% (29)	0.7633	0.0677
CNI (Cyclosporine A or Tacrolimus)	100% (36)	100% (36)	> 0.9999	0
Mycophenolate	97.2% (35)	97.2% (35)	> 0.9999	0
Graft function at first outpatient visit				
Serum creatinine	1.981 ± 0.1502	2.146 ± 0.2310	0.5516	0.132
eGFR CKD‐EPI (creatinine)	44.11 ± 3.623	43.52 ± 3.774	0.9101	0.0271

*Note:* % (*n*) or mean ± SEM, *t*‐tests with Welch's correction. SMDs are given for all covariates after matching.

These data are consistent with an association of frequent UTI and cardiovascular disease in a high‐risk human population.

### 3.2 Pyelonephritis Enhances Atherosclerotic Plaque Necrotic Core Formation

**FIGURE 1:**
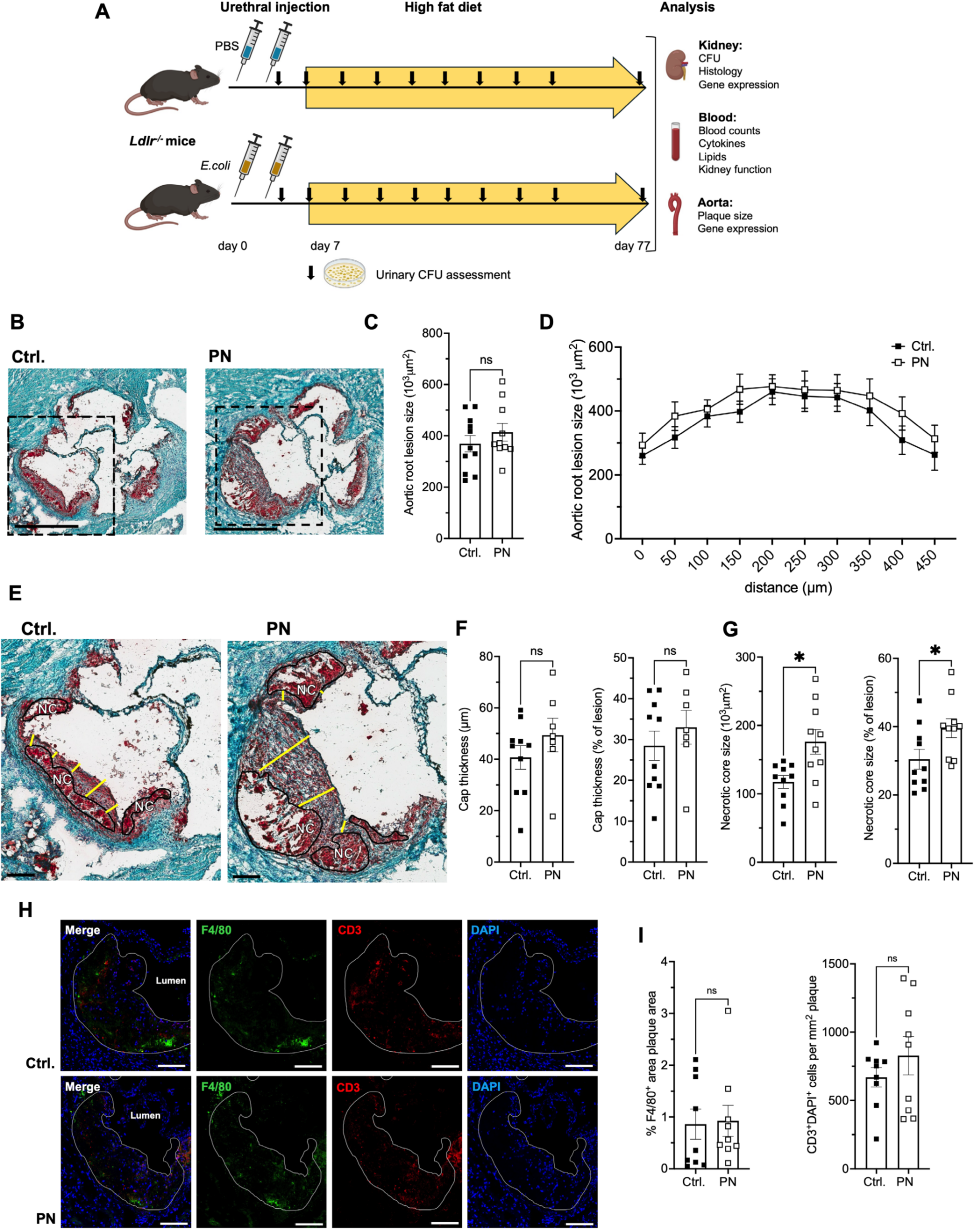
Pyelonephritis increases atherosclerotic plaque necrotic core size. (A–H) Atherosclerosis was promoted by a high‐fat diet for ten weeks, starting one week after induction of pyelonephritis (PN) in female *Ldlr* ^*−/−*^ mice (experimental setup in A). (B–I) Aortic root lesions were assessed in mice that cleared kidney infection in the kidneys and urine. Total lesion size (C, D serial sections), fibrotic cap thickness (E, F), and the proportion of necrotic cores (E, G) were enumerated (B, E: Examples of Oil‐Red‐O stained sections, NC: Necrotic core, yellow lines: Cap thickness measurements, B, C, E, G: Statistical analyses: *N* = 7–11 from 5 indep. experiments, *t*‐tests with Welch's correction, ANOVA (C)). (H, I) Immunostaining for macrophage marker F4/80 and T cell marker CD3 was performed with nuclear counterstain (H examples, I statistical analysis of *n* = 9 per group, *t*‐tests with Welch's correction) (Scale bars indicate 500 μm (B) and 100 μm (E, H)).

**FIGURE 2:**
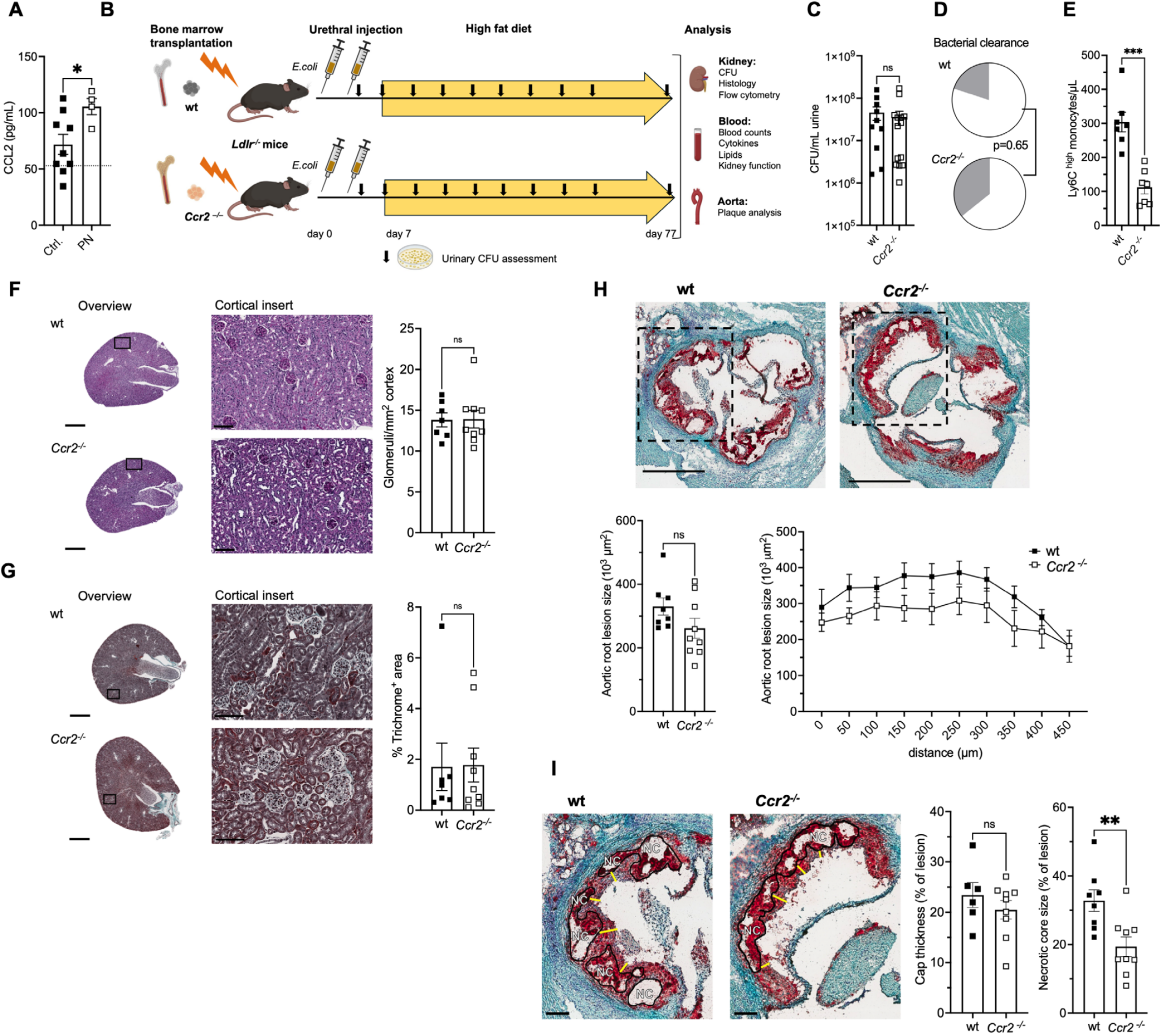
Myeloid CCR2 mediates increased atherosclerotic lesion necrotic core after pyelonephritis. (A) Serum CCL2 was analyzed with a cytometric bead assay 4 weeks after infection (PN) (*n* = 4–9 in 3 indep. exp.; *t*–test with Welch's correction). (B–I) *Ldlr* ^*−/−*^ mice were lethally irradiated and reconstituted with *Ccr2* ^*−/−*^ or control wildtype bone marrow (BM) and sacrificed after ten weeks of a high‐fat diet starting one week after induction of pyelonephritis (PN) (experimental setup in B). (C) Initial urinary CFU counts (*n* = 10–14/group in 5 indep. exp.; *t*‐tests with Welch's correction). (D) Bacterial clearance rates combined for urine and kidney at the end of the experiment (white indicates clearance, *n* = 10–14, 5 indep. exp.; Fisher's exact test *p* = 0.6529). (E) Circulating inflammatory monocyte counts (*n* = 8–9/group in 5 indep. exp.; *t*‐tests with Welch's correction). (F, G) Renal histology in mice without residual infection was assessed after PAS (F) and trichrome staining for fibrosis assessment (G) (examples, size bars indicate 1 mm and 100 μm, and quantification in = 7–9, 5 indep. transplantations, *t*–tests with Welch's correction). (H–I) Aortic root lesions were assessed in mice without residual infection. Total lesion size (H). serial section, fibrotic cap thickness, and peak lesion necrotic core size (I) were enumerated (examples, NC: Necrotic core, yellow lines: Cap thickness measurements, scale bars indicate 500 μm and 100 μm, statistical analyses of *n* = 6–9 from 5 indep. transplantations, *t*–tests with Welch's correction, and ANOVA).

To investigate mechanisms by which acute PN affects atherosclerotic lesion formation, female *Ldlr* ^*−/−*^ mice were infected twice transurethrally with uropathogenic *E. coli* to induce kidney infection as described [30, 33] (Figure 1A). Urinary bacterial clearance was assessed by serial bacterial colony‐forming units (CFU) measurements as indicated. In addition, kidney CFU were tested at sacrifice, when the kidneys were also assessed for macroscopic and histological evidence of persisting infection [30, 31]. The present analysis addresses atherosclerosis development in mice that cleared the infection. As reported, and similar to other investigations on kidney recovery after a single infection episode in mice [37, 38], renal histology and cortical glomerular density as a measure of nephron number were well preserved in animals that cleared the infection (Figure 2) [31].

Clinical assessment revealed no differences in body, spleen, or kidney weights (Table S1). Also, full blood counts and kidney functional markers, serum urea, and creatinine did not differ from those of otherwise identically treated sham‐infected animals. There were no significant differences in serum cholesterol or triglyceride levels (Table S1).

Atherosclerotic lesion size was measured after ten weeks on a high‐fat diet. Total lesion size did not differ between mice after PN and controls (Figure 1B–D). Absolute and relative cap thickness were unaltered (Figure 1E,F), but both relative and absolute necrotic core size increased significantly (Figure 1E,G). Lesional macrophage and T cell densities did not detectably differ (Figure 1H,I). The increase in necrotic core size is suggestive of lesion instability after pyelonephritis.

### 3.3 Bone Marrow CCR2 Is Not Required for Host Response, but Promotes Atherosclerotic Plaque Necrosis After Pyelonephritis

**FIGURE 3:**
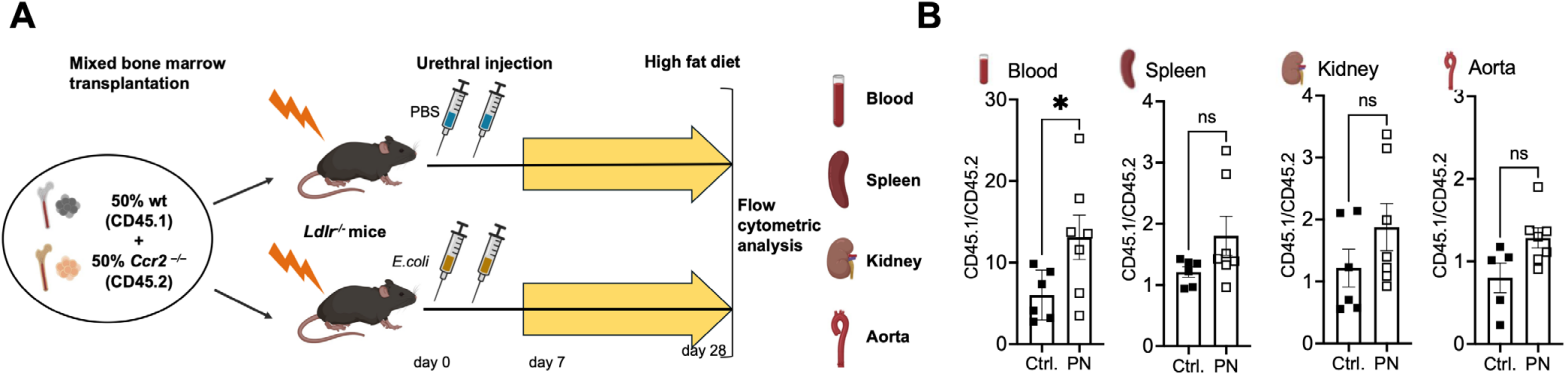
CCR2 mediates monocyte mobilization during atherosclerotic lesion formation after pyelonephritis. (A, B) *Ldlr* ^*−/−*^ mice were lethally irradiated and reconstituted with a 1:1 mixture of wildtype (wt, CD45.1) or *Ccr2* ^*−/−*^ (CD45.2) bone marrow (BM) before pyelonephritis (PN) induction, and monocyte and myeloid cell distribution was analyzed after three weeks on a high‐fat diet (experimental outline in A). (B) Blood CD115^+^ monocytes and splenic, renal, and aortic CD11b^+^ myeloid cells were assessed by flow cytometry according to the genotype identified by CD45.1 and CD45.2 congenic markers (gating in Figure 6). *n* = 5–7, two indep. transplantations, *t*–test with Welch's correction.

To analyze systemic pro‐inflammatory mediators after PN, cytometric bead arrays were employed. Early during atherosclerotic lesion formation, after three weeks of a high‐fat diet and four weeks after PN induction, monocyte chemoattractant CCL2 was significantly elevated in serum (Figure 2A). The other studied mediators were unaltered (Suppl. Figure 3A). After eleven weeks, significant differences in systemic cytokine levels were no longer detected (Figure 3B).

**FIGURE 4:**
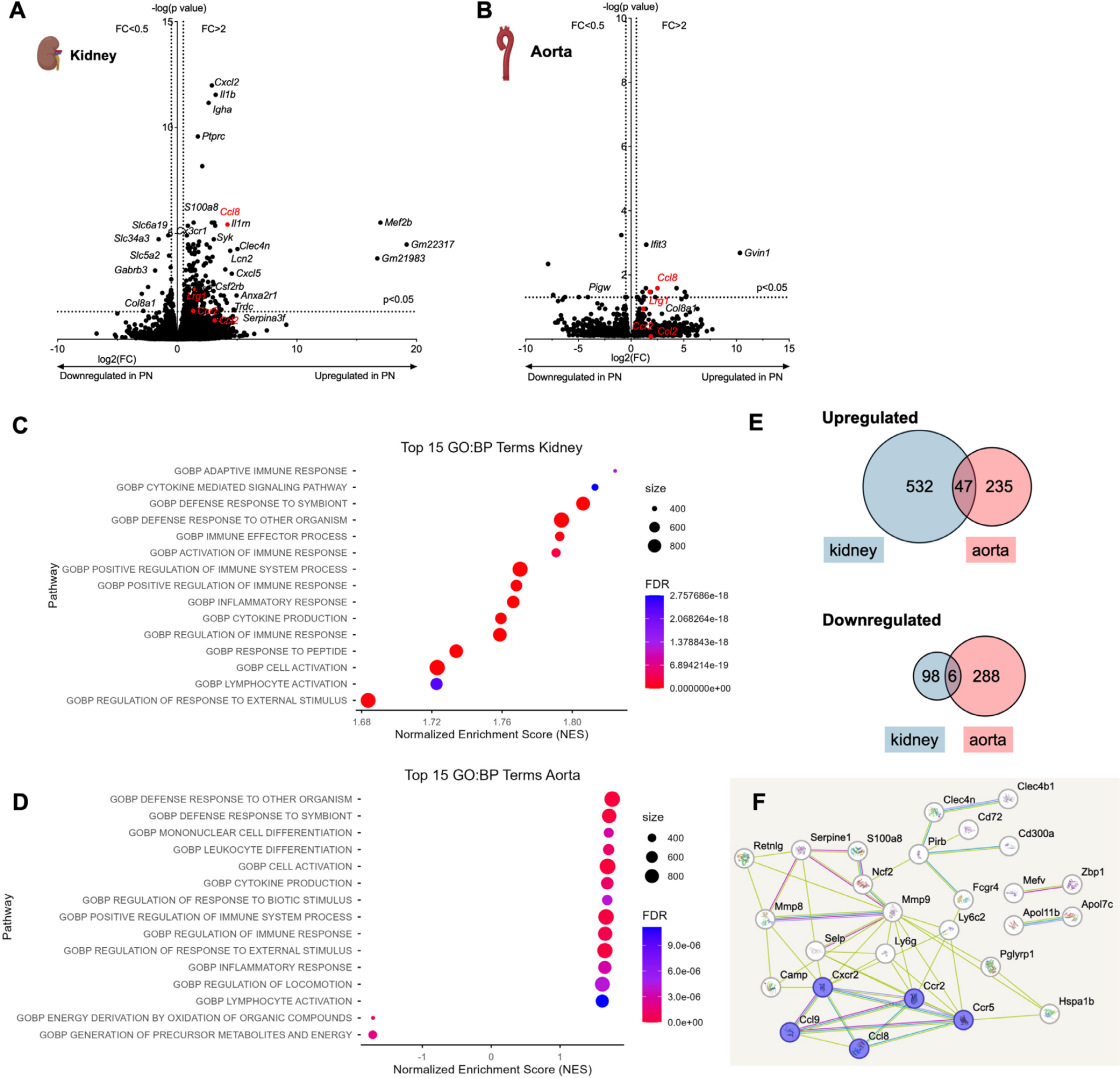
Increased inflammatory gene expression during atherosclerotic lesion formation after pyelonephritis. (A–F) Atherosclerosis was promoted by a high‐fat diet for three weeks, starting one week after pyelonephritis (PN) induction in female *Ldlr* ^*−/−*^ mice. Renal and aortic gene expression were studied by single‐cell sequencing (*n* = 5–6 per group). (A, B) Volcano plots of regulated genes in the kidney (A) and aorta (B) compared to the control (dotted lines indicate a corrected *p*‐value < 0.05 and twofold increase or decrease). (C, D) The top 15 significantly enriched GO terms for biological processes (padj < 0.05) identified by gene set enrichment analysis for kidney (C) and aorta (D) are shown. (E) Overlap of upregulated and downregulated genes between mouse kidney and aorta RNA‐seq datasets (E, raw *p*‐value < 0.05 with log2FC±1 threshold). (F) STRING network analysis on the shared, connected upregulated genes (chemokines and receptors marked in purple).

To test for a functional role for in increased necrotic core size after PN, CCL2 receptor CCR2 was ablated by replacing the bone marrow of *Ldlr* ^*−/−*^ mice with 100% *Ccr2* ^*−/−*^ or control wildtype bone marrow (experimental outline in Figure 2B). Replacement efficacy as evaluated by flow cytometric analysis of congenic markers CD45.1/CD45.2 was 79.1% ± 8.8% (*n* = 5 per group, 2 indep. transplantations) among blood monocytes in agreement with ablation of myeloid CCR2 surface protein and bone marrow *Ccr2* mRNA expression (Figure 4). Mice were subjected to a PN episode followed by ten weeks of a high‐fat diet (Figure 2B). Initial urine bacterial load and subsequent clearance rates did not differ between the bone marrow genotypes (Figure 2C,D). Mice that cleared the infection were included in subsequent analyses. Clinical, hematologic, and serum chemistry parameters did not differ significantly, with the exception of total and inflammatory blood monocyte counts that were expectedly lower in the absence of bone marrow *Ccr2* as described [22] (Table 3, Figure 2E). Also, plasma CCL2 levels were expectedly higher in the absence of CCR2 [39, 40] (Table 3). Kidney histologic analysis revealed no difference in cortical glomerular density or fibrosis development (Figure 2F,G). Renal flow cytometry showed very similar total leukocyte and macrophage counts at the end of the experiment, i.e., eleven weeks after infection (Suppl. Figure 5). Macrophage subtype distribution and surface marker F4/80 and MHC II expression also did not differ significantly between the genotypes.

Atherosclerotic aortic root lesion size in mice with wild‐type and *Ccr2* ^*−/−*^ bone marrow was very similar (Figure 2H). While relative cap thickness was unaltered, necrotic core size was significantly lower in the absence of bone marrow *Ccr2* (Figure 2I), suggestive of a mechanistic function of *Ccr2* in plaque necrotic core size growth after PN.

To investigate the underlying mechanism, mixed bone marrow chimeric *Ldlr* ^*−/−*^ mice with 50% wildtype and 50% *Ccr2* ^*−/−*^ bone marrow were generated. After PN and subsequent atherosclerosis induction, flow cytometry was conducted after three weeks on a high‐fat diet to assess infiltration in early stages of plaque formation (Figure 3A). Leukocytes were distinguished by the congenic markers (CD45.1 (wildtype) and CD45.2 (*Ccr2* ^*−/−*^)) (gating strategies in Figure 6). In the absence of *Ccr2*, blood monocytes significantly decreased compared to wild‐type cells in an identical environment (Figure 3B). In contrast, spleen, kidney, and aortic macrophage numbers were not significantly affected by *Ccr2* on an individual cell level.

These data propose that myeloid *Ccr2*, while without detectable impact on renal antibacterial host response, mechanistically mediates plaque necrosis in the atherosclerotic aorta after PN by enhancing monocyte mobilization into the circulation.

### 3.4 Pyelonephritis Alters Aortic Gene Expression During Atherogenesis

To assess renal and aortic PN effects during atherosclerosis development in a non‐hypothesis‐driven approach, kidney and aortic transcriptome analyses were performed after three weeks on a high‐fat diet, four weeks after the PN episode. Clinical parameters were very similar between the groups also at this time point (Table 2). Kidney histologic analysis revealed no differences in glomerular density or fibrosis staining (Figure 7). Atherosclerotic lesions were expectedly small. No significant differences between the groups were detected (Figure 8).

RNA sequencing demonstrated a marked upregulation of innate and adaptive immune response genes in the kidney, which was replicated in the aorta to a lesser degree (Figure 4A–D). In an exploratory approach, genes up‐ or downregulated at least twofold with a raw *p* < 0.05 were explored for overlap between the organs. 47 commonly up‐ and 6 commonly downregulated genes were present (Figure 4E, full list inSuppl. Table 4). In the upregulated group, STRING analysis illustrates mainly innate immune genes, including chemokines related to CCR2 (Figure 4F). With a more restrictive approach, employing a corrected *p*‐value < 0.05, two genes, *Ccl8* and *Lrg1*, overlapped. Among them, CCL8 mRNA, a human CCR2 ligand, was regulated most markedly in both kidney and aorta (2.8 fold); LRG1 mRNA was upregulated 1.8 fold (Figure 4A,B). The murine CCL8‐receptor *Ccr8* was below the detection limit (data not shown).

These data depict a sustained local and aortic inflammatory response with chemokine pathway upregulation during atherogenesis after pyelonephritis.

### 3.5 Regulation of Pyelonephritis‐Responsive Aortic Genes in Human Atherosclerotic Plaques

**FIGURE 5:**
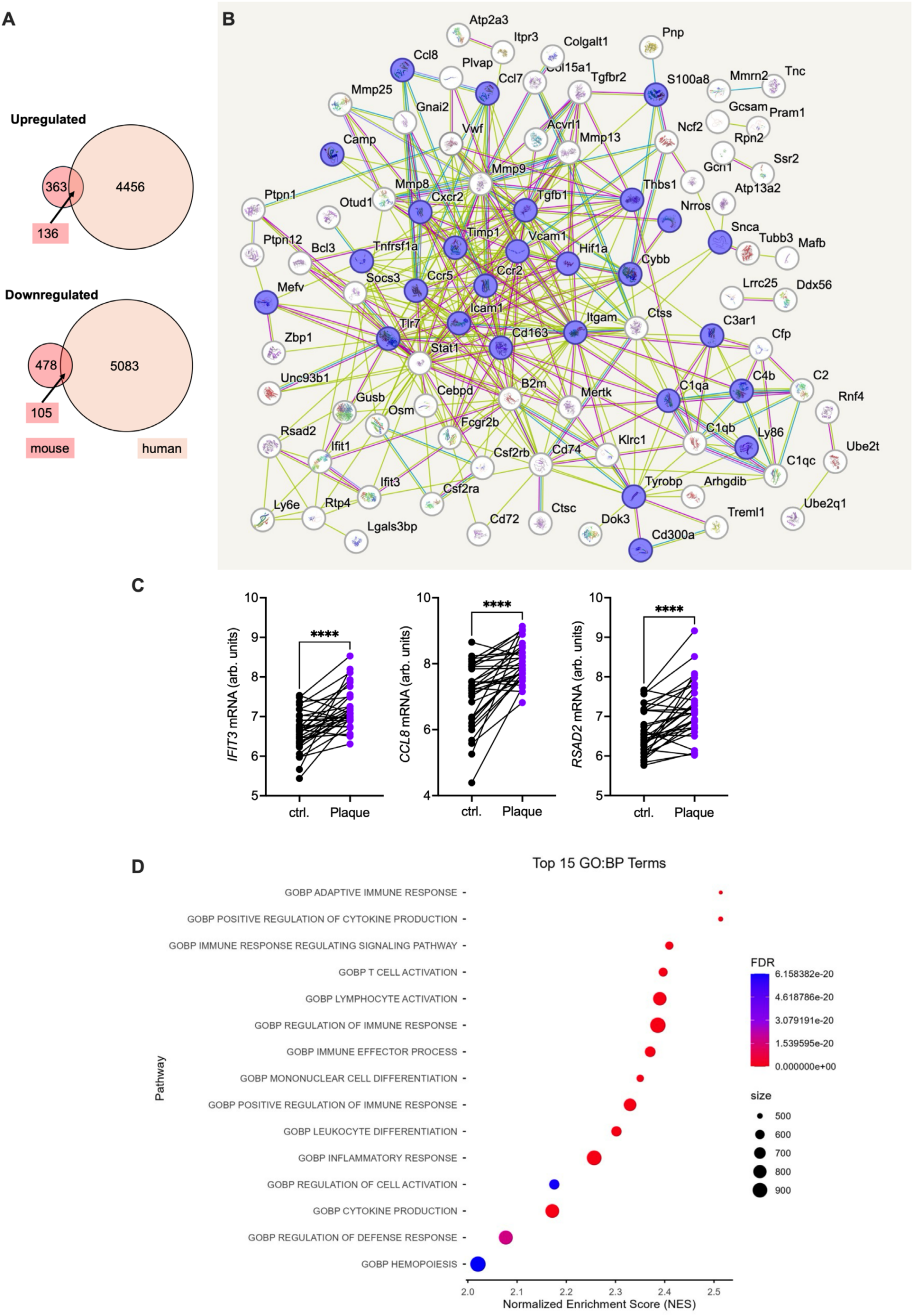
CCL8 defines an inflammatory environment in the human atherosclerotic plaque. (A–D) Genes regulated in the murine aorta after pyelonephritis compared to otherwise identically treated control *Ldlr* ^*−/−*^ animals were compared to gene regulation in human atherosclerotic plaques compared to macroscopically intact arteries from 32 donors (GSE43292). (A) Overlap of individual up‐ and downregulated genes with a raw *p*‐value < 0.05. (B) Visualization of upregulated connected genes in both settings by STRING (chemokines and receptors marked in purple). (C) Expression of the genes with a corrected *p* < 0.05 in both comparisons of human atherosclerotic plaque (paired *t*‐tests). (D) Top 15 significantly enriched GO Biological Process terms (padj < 0.05) identified by gene set enrichment analysis in comparison of human atherosclerotic plaque samples with *CCL8* above and below the median.

To start to explore the human relevance of our findings, genes upregulated in the murine aorta in atherosclerosis after PN were compared to gene regulation in human atherosclerotic plaques compared to macroscopically unaffected tissue from the same donors [41] (GSE43292). In an exploratory approach to detect possibly regulated functional gene groups, genes regulated with a raw *p*‐value < 0.05 were compared. Among up‐ and downregulated genes, overlap was found, mainly among innate immune and chemokine genes (Figure 5A–C). With a more restrictive approach, employing a corrected *p*‐value < 0.05, three genes, namely interferon type 1 signaling genes *IFIT3* and *RSAD2*, and human CCR2 ligand *CCL8*, showed upregulation in human plaques in parallel to the murine situation (Figure 5C). To start to further address the plaque environment in relation to their *CCL8* expression, human plaques with *CCL8* above and below the median were compared. Higher *CCL8* mRNA expression is associated with an inflammatory and adaptive immune response signature (Figure 5D).

### 3.6 CCL8 Increases in Human Pyelonephritis and Promotes Human Monocytes Chemotaxis

Data on CCL8 function and regulation are limited, especially in humans. We therefore studied its regulation in human PN. CCL8 became detectable, different from control individuals, while CCL2 plasma levels did not change significantly (Figure 9). This extends a published observation in gram‐positive sepsis [42].

**FIGURE 6:**
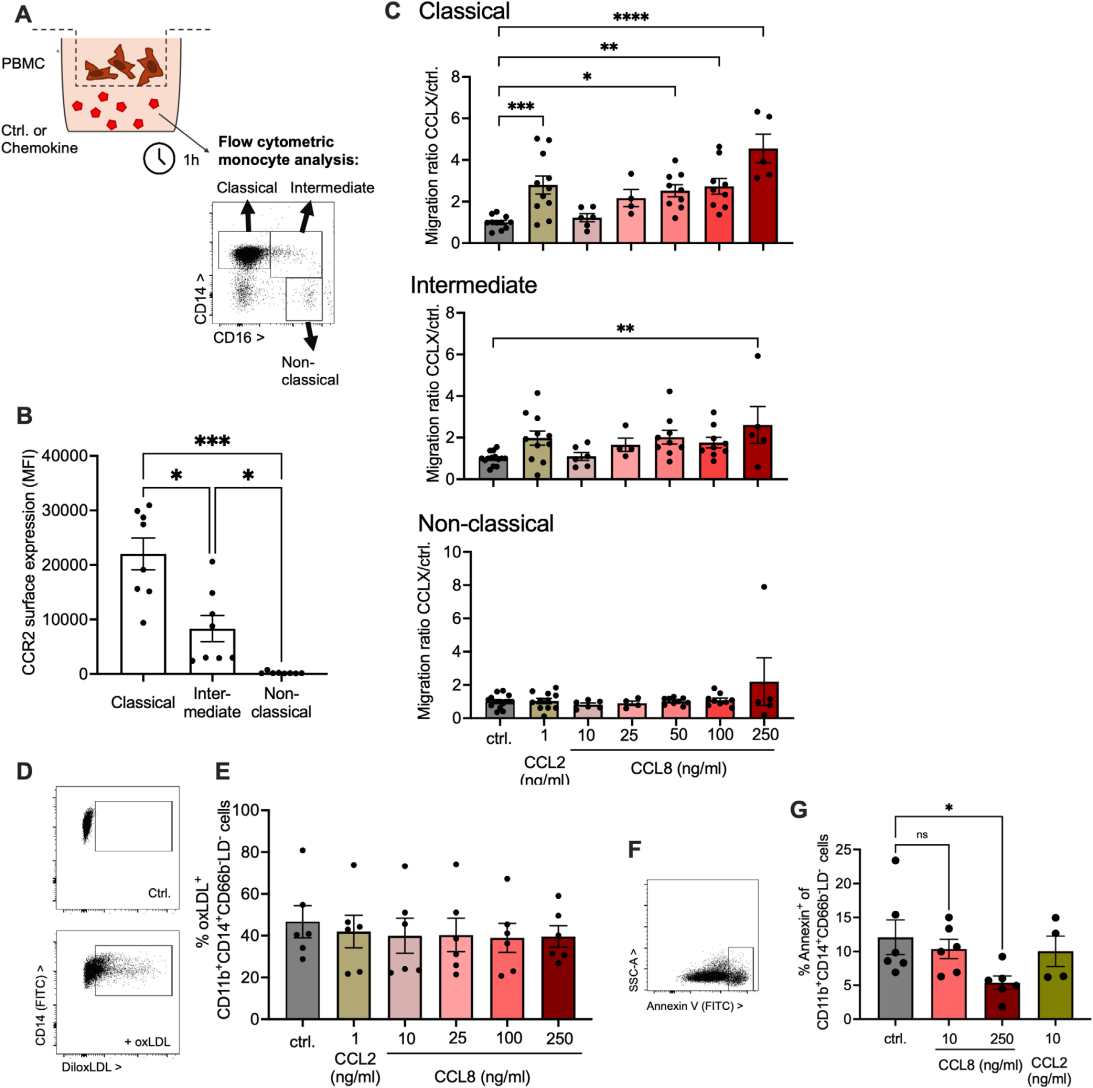
CCL8 promotes human monocyte migration and survival. (A–C) Human primary monocyte migration towards CCL2 and CCL8 was studied by flow cytometry separately for classical (CD14^+^CD16^−^), intermediate (CD14^+^CD16^+^), and non‐classical (CD14^−^CD16^+^) subsets (experimental setup in A). (B) CCR2 surface expression according to subset (*n* = 8 donors, Tukey's after ANOVA). (C) Quantification of cell migration relative to unstimulated samples (*n* = 4–11 donors in 2–6 indep. exp.; Dunnett's after ANOVA). (D, E) Uptake of Dil‐labeled oxidized LDL after 3 h was assessed by flow cytometry (*n* = 6 donors in 3 indep., ANOVA not significant). (F, G) Apoptosis was assessed by Annexin V binding (*n* = 6 donors in 3 indep., Dunnett's after ANOVA, CCL2 serving as control).

The role of CCL8 in human monocyte chemotaxis was investigated in primary cells in vitro and compared to CCL2, as a potent human CCR2 ligand [43] (Figure 6A–C). Their receptor CCR2 was mainly expressed on classical and, to some degree, on intermediate cells as described [44](Figure 6B). Both significantly induced classical monocyte migration (Figure 6C) as originally described for total PBMC in similar doses [45, 46]. It did not induce migration of other subsets. OxLDL uptake and macrophage polarization markers were not measurably affected (Figure 6D,E, Figure 10). CCL8, but not CCL2, prevented constitutive human classical monocytes' apoptosis (Figure 6F,G). CCL2 remained inert in this regard also at the maximal CCL8 dose of 250 mg/dL (ctrl: 5.8% ± 3%, CCL2: 6.7% ± 2%, *n* = 6 donors in 3 experiments, *p* = 0.62).

Increased migration and decreased cell death may contribute to the promotion of an unstable plaque phenotype by CCL8.

## 4 Discussion

Our results demonstrate an unstable plaque atherosclerotic phenotype after an episode of pyelonephritis and propose inflammatory monocytes as underlying pathogenic mediators. Atherosclerotic plaque gene regulation after PN substantially overlapped with regulation in human atherogenesis, namely regarding innate immune genes and elevation of CCR2‐ligand CCL8.

With our murine experiments, we established an in vivo model of atherosclerosis after PN. A preceding PN episode increased relative and absolute necrotic core formation in the aortic root without changing overall plaque size. With all due caution regarding parallels of human and murine plaque vulnerability [47], this may indicate increased instability and propensity towards cardiovascular events.

Systemic cytokine assessment suggested CCL2 as a potential systemic inflammatory mediator after PN. Indeed, our bone marrow‐chimera studies establish chemokine receptor CCR2 as a mediator of this phenotype. After PN, CCL2 mediated monocyte mobilization to the blood. This differs from another renal insult, namely ischemia–reperfusion injury, where monocytes home to the kidney CCR2‐dependently [29]. Both kidney insults promoted atherosclerosis. These data also underline the importance to address specific diseases to adequately design possible interventions. Indeed, during the host response in experimental PN in our experimental setup, CCR2 was dispensable. This agrees with earlier data from other groups [37, 48], which is central if therapeutic interventions are to be considered. Also, human carriers of a deficient allele are protected from atherosclerosis, but not clinically prone to infection [24]. Regarding atherosclerosis therapies, pharmacologic interventions to block CCR2 in murine models yielded mixed results [49, 50, 51, 52], albeit in the absence of kidney injury.

We broadened our search for underlying mechanisms of kidney‐aorta crosstalk by profiling the murine atherosclerotic aortas via gene expression screen. While further detailed studies of leukocyte composition and protein expression within the atherosclerotic plaques after pyelonephritis are warranted, gene expression analysis revealed upregulation of adaptive and innate immune and inflammatory genes not only in the kidney, but also in the atherosclerotic aorta four weeks after PN. Monocyte genes, most notably CCL8, were upregulated in both organs. To investigate human relevance, we tested whether human plaques show enrichment of genes regulated in murine aortas after PN. Among genes upregulated in both, innate immune and chemokine genes were prominent. A markedly regulated gene in this regard was again CCL8, a human CCR2 ligand and monocyte chemoattractant. Our data in primary human monocytes delineate that it significantly promotes only CCR2^+^ monocyte migration, extending an early general monocyte study [45]. Our experiments also determine that CCL8, while without a detectable effect on oxLDL uptake, supports their survival, which may further contribute to a pro‐atherogenic function. Further studies are needed to determine how CCL8 is regulated in the plaque during atherogenesis and whether it mechanistically impacts its stability, keeping in mind its differential roles in human and murine systems [53, 54, 55]. CCL8 became detectable in human plasma in our human PN cohort, potentially enabling pathophysiologic relevance.

UTI is associated with cardiovascular events in large population‐based observations [6, 7, 8, 9, 10]. We add to these data by investigating a cohort with a high incidence of both kidney transplant recipients. Our data show elevated cardiovascular event rates in patients with UTI above the median, also in this group. While our analysis is limited by a moderate cohort size and its cross‐sectional design precluding analysis of endpoints such as death or graft loss, it extends previous reports to an immunosuppressed population with a chiefly T‐cell targeted therapy, i.e., calcineurin inhibitors as a mainstay of therapy. Adaptive immunity only plays a minor role in antibacterial response in the urinary tract [19, 20]. However, these patients' host defense will rely even more on innate immunity than in other populations. The pathophysiologic relevance of monocytes in mediating plaque necrosis after PN in the murine model would therefore conceivably be of significant relevance also to this patient population.

Taken together, our data delineate CCR2‐mediated monocyte mobilization as a mediator of altered plaque structure after pyelonephritis. They introduce CCL8 as a marker of inflammatory plaques and a modulator of human inflammatory monocyte survival in addition to migration. The results may aid the design of targeted therapies in the peri‐infectious high‐risk period for cardiovascular events.

## Author Contributions

L.P., P.F., E.K.K., and S.V. designed research, L.P., P.F., M.F., and J.M. conducted experiments, L.R.‐S., U.S., and F.W. recruited patients, C.K. provided reagents, L.P., P.F., M.F., G.W.S., and S.V. analyzed data, L.P., P.F., and S.V. wrote the manuscript with help from all coauthors, and all authors read and approved the manuscript.

## Funding

C.K., F.W., and S.v.V. are supported by German Research Foundation FOR 5427/1 (466 687 329), and C.K. and S.v.V. by German Research Foundation SFB1192 (264 599 542) and members of the Excellence‐Cluster “Immunosensation^3^” (EXC2151), which is funded as an institution by DFG (390873048). S.v.V. is supported by the German Research Foundation (DFG) (450 775 971).

## Conflicts of Interest

The authors declare no conflicts of interest.

## Supporting information


**TABLE S1:** Characteristics of Ldlr^−/−^ mice after 10 weeks high‐fat diet.
**Table S2:** Characteristics of Ldlr^−/−^ mice after 3 weeks high‐fat diet.
**Table S3:** Ldlr^−/−^ mice after reconstitution with wild‐type or Ccr2^−/−^ bone marrow and 10 weeks of a high‐fat diet.
**Table S4:** Parallel gene regulation in the kidney and aorta 4 weeks after pyelonephritis.
**Figure S1:** Covariate balance after propensity score matching. Standardized mean differences (SMD) and Kolmogorov–Smirnov test results for each variable before (blue circles) and after (red triangles) propensity score matching (PSM) are shown.
**Figure S2:** Kidney histology in Ldlr^−/−^ mice 11 weeks after pyelonephritis. (A, B) Atherosclerosis was promoted by a high‐fat diet for 10 weeks, starting 1 week after induction of pyelonephritis (PN) in female *Ldlr* ^*−/−*^ mice. A: Representative PAS‐stained sections are shown size bars indicate 1 mm in overview and 100 μm in cortical examples. B: Quantification in *n* = 9–12 mice in 7 indep. exp.; *t*‐tests with Welch's correction.
**Figure S3:** Systemic cytokine levels during atherosclerosis induction after pyelonephritis. (A. B) In Ldlr^−/−^ mice treated as described in Figure 1A, serum TNFα, IFNγ, IL‐6, IL‐10, and CCL2 were analyzed with a cytometric bead assay 4 (A) and 11 weeks after kidney infection (PN) (A: *n* = 4–9. 3 indep. exp.; B: *n* = 9–12.7 indep. exp.; *t*‐tests with Welch's correction. Dotted lines indicate detection limit).
**Figure S4:** CCR2 assessment in complete bone marrow chimeric Ldlr^−/−^ mice. (A–D) Ldlr^−/−^ mice reconstituted with Ccr2^−/−^ or control wild‐type bone marrow were sacrificed after 10 weeks of a high‐fat diet starting 1 week after induction of pyelonephritis as depicted in Figure 2B. (A–C) Assessment of bone marrow (B) and blood (C) myeloid CCR2 surface expression (A: gating strategy; B, C: statistical analysis of *n* = 4–6. 3 indep. transplantations. *t*‐tests with Welch's correction). (D) Renal Ccr2 mRNA expression was assessed by qPCR (*n* = 7–8 from 4 indep. transplantations. *t*‐test with Welch's correction. A Ccr2^−/−^ mouse serving as negative control).
**Figure S5:** Kidney macrophage characterization after PN in the absence and presence of Ccr2. (A–C) Ldlr^−/−^ mice were lethally irradiated and reconstituted with Ccr2^−/−^ or control wild‐type bone marrow (BM) and sacrificed after 10 weeks of a high‐fat diet starting 1 week after induction of pyelonephritis (PN) as outlined in Figure 2B. Renal leukocyte enumeration by flow cytometry (gating strategy (A), statistical analysis frequencies (B), and mean surface expression levels (C) of *n* = 8–9/group in 5 indep. exp.; *t*‐tests with Welch's correction).
**Figure S6:** Flow cytometric gating strategy for the assessment of mixed bone marrow chimeras. (A–D) Gating strategies for blood monocytes (A) and spleen (B), kidney (C) and aortic (D) leukocytes for CD11b^+^ myeloid cells and CD45.1 and CD45.2 congenic markers are shown.
**Figure S7:** Kidney histology in Ldlr^−/−^ mice 4 weeks after pyelonephritis. (A–D) Atherosclerosis was promoted by high‐fat diet for 3 weeks starting 1 week after induction of pyelonephritis (PN) in female Ldlr^−/−^ mice and renal histology assessed after PAS (A, B) and trichrome staining for fibrosis assessment (C, D) (A, C: Examples. Size bars indicate 1 mm and 100 μm. B, D: Quantification in *n* = 6–11 mice in 4 indep. exp.; *t*‐tests with Welch's correction).
**Figure S8:** Aortic lesion size in Ldlr^−/−^ mice after 3 weeks of a high‐fat diet and 4 weeks after pyelonephritis. Atherosclerosis was promoted by a high‐fat diet for 10 weeks starting 1 week after induction of pyelonephritis (PN) in female Ldlr^−/−^ mice. Examples of Oil‐red‐O‐stained aortic roots and results of statistical analysis are shown (size bars indicate 500 μm. *n* = 6–11 mice in 4 indep. Exp.; *t*‐test with Welch's correction).
**Figure S9:** Systemic CCL2 and CCL8 regulation in human pyelonephritis. (A, B) Plasma from patients with acute pyelonephritis (PN) was assessed by ELISA for CCL2 (D) and CCL8 (E; *n* = 7 ctrl. and 30 PN). One‐sided test of CCL8 detection. CCL2: *t*‐test with Welch's correction.
**Figure S10:** Impact of CCL2 and CCL8 on human monocyte‐derived macrophage differentiation. (A, B) Human primary monocyte‐derived macrophages were stimulated with 100 ng/mL CCL2 or CCL8 during 7 days of differentiation. Surface expression of M1 type markers CD64 and HLA‐DR, APC type marker CD86, M2 type marker CD206, and CD163 U(hemoglobin scavenger) was assessed by flow cytometry (Gating in A, B: mean fluorescence intensities (MFIs) or the indicated markers from *n* = 6 donors in 3 indep. exp.; ANOVAs not significant).

## Data Availability

Gene expression data is available as GSE313813 at https://www.ncbi.nlm.nih.gov/geo/query/acc.cgi?acc=GSE313813. All other data is available upon reasonable request.
