# Supplementary material for: Monocyte Chemokines Enhance Atherosclerotic Plaque Necrosis After Bacterial Kidney Infection
Source: FASEB J. 2026 Apr 3;40(7):e71720. doi: 10.1096/fj.202505052R (PMC13047719; doi:10.1096/fj.202505052R)
Supplement: Supplementary file 1 — TABLE S1: Characteristics of Ldlr−/− mice after 10 weeks high‐fat diet. Table S2: Characteristics of Ldlr−/− mice after 3 weeks high‐fat diet. Table S3: Ldlr−/− mice after reconstitution with wild‐type or Ccr2−/− bone marrow and 10 weeks of a high‐fat diet. Table S4: Parallel gene regulation in the kidney and aorta 4 weeks after pyelonephritis. Figure S1: Covariate balance after propensity score matching. Standardized mean differences (SMD) and Kolmogorov–Smirnov test results for each variable before (blue circles) and after (red triangles) propensity score matching (PSM) are shown. Figure S2: Kidney histology in Ldlr−/− mice 11 weeks after pyelonephritis. (A, B) Atherosclerosis was promoted by a high‐fat diet for 10 weeks, starting 1 week after induction of pyelonephritis (PN) in female Ldlr −/− mice. A: Representative PAS‐stained sections are shown size bars indicate 1 mm in overview and 100 μm in cortical examples. B: Quantification in n = 9–12 mice in 7 indep. exp.; t‐tests with Welch's correction. Figure S3: Systemic cytokine levels during atherosclerosis induction after pyelonephritis. (A. B) In Ldlr−/− mice treated as described in Figure 1A, serum TNFα, IFNγ, IL‐6, IL‐10, and CCL2 were analyzed with a cytometric bead assay 4 (A) and 11 weeks after kidney infection (PN) (A: n = 4–9. 3 indep. exp.; B: n = 9–12.7 indep. exp.; t‐tests with Welch's correction. Dotted lines indicate detection limit). Figure S4: CCR2 assessment in complete bone marrow chimeric Ldlr−/− mice. (A–D) Ldlr−/− mice reconstituted with Ccr2−/− or control wild‐type bone marrow were sacrificed after 10 weeks of a high‐fat diet starting 1 week after induction of pyelonephritis as depicted in Figure 2B. (A–C) Assessment of bone marrow (B) and blood (C) myeloid CCR2 surface expression (A: gating strategy; B, C: statistical analysis of n = 4–6. 3 indep. transplantations. t‐tests with Welch's correction). (D) Renal Ccr2 mRNA expression was assessed by qPCR (n = 7–8 from 4 indep. transplantations. [file FSB2-40-e71720-s001.pdf]

Supplemental material

Monocyte chemokines enhance atherosclerotic plaque necrosis after bacterial kidney infection

Suppl. tables 1-4

Suppl. figures 1-10

Suppl. Tables

Suppl. table 1: Characteristics of Ldlr$^{-/-}$ mice after ten weeks high fat diet

	Ctrl.	Pyelonephritis	p-value
**Clinical characteristics**			
Body weight (g)	24.3±0.6 (12)	23.6±0.8 (10)	0.53
Spleen weight (mg)	155.4±8.3 (12)	148.0±6.5 (10)	0.49
Rel. spleen weight (%)	0.6±0.03 (12)	0.6±0.02 (10)	0.72
Right kidney weight (mg)	175.7±10.5 (12)	175.0±11.5 (10)	0.51
Rel. right kidney weight (%)	0.7±0.04 (12)	0.7±0.03 (10)	0.80
**Hematological values**			
Total leukocytes (10^3/µl)	8.2±1.3 (9)	9.1±1.3 (8)	0.65
Monocytes (10^3/µl)	0.3±0.07 (9)	0.4±0.04 (8)	>0.99
Lymphocytes (10^3/µl)	5.4±0.9 (9)	5.8±0.7 (8)	0.69
Granulocytes (10^3/µl)	2.5±0.4 (9)	2.9±0.5 (8)	0.66
Thrombocytes (10^3/µl)	841.7±146.6 (9)	859.3±108.0 (8)	0.62
Erythrocytes (10^6/µl)	11.1±0.4 (10)	10.0±1.1 (8)	0.32
Hemoglobin (g/dl)	17.1±0.9 (10)	15.6±1.9 (8)	0.50
Hematocrit (%)	59.8±2.1 (10)	52.8±5.7 (8)	0.29
**Clinical chemistry values**			
Urea (mg/dl)	54.26±3.6 (5)*	57.9±3.3 (7)*	0.47
Creatinine (mg/dl)	0.36±0.1 (4)*	0.45±0.1 (6)*	0.30
Cholesterol (mg/dl)	1476±113.2 (6)$^+$	1730±26.19 (5)$^+$	0.07
Triglycerides (mg/dl)	637.2±63.8 (6)$^+$	711.2±51.1 (5)$^+$	0.39

Values are mean±SEM (n). T-tests with Welch's correction. *pooled from n=15-16/group. $^+$pooled from 10-12/group

Suppl. table 2: Characteristics of Ldlr$^{-/-}$ mice after three weeks high fat diet

	**Ctrl.**	**Pyelonephritis**	**p-value**
**Clinical characteristics**			
Body weight (g)	20.5±0.5 (13)	20.8±0.6 (7)	0.73
Spleen weight (mg)	124.4±4.3 (13)	120.2±7.3 (7)	0.63
Rel. spleen weight (%)	0.6±0.01 (13)	0.6±0.04 (7)	0.56
Right kidney weight (mg)	164.6±4.4 (13)	159.9±2.6 (7)	0.38
Rel. right kidney weight (%)	0.8±0.02 (13)	0.7±0.01 (7)	0.15
**Hematological values**			
Total leukocytes (10^3/µl)	11.8±0.9 (10)	12.5±2.7 (3)	0.81
Monocytes (10^3/µl)	0.5±0.05 (10)	0.5±0.1 (3)	0.94
Lymphocytes (10^3/µl)	7.9±0.6 (10)	8.1±1.6 (3)	0.95
Granulocytes (10^3/µl)	3.2±0.3 (10)	3.9±1.1 (3)	0.62
Thrombocytes (10^3/µl)	871.3±118.7 (10)	859.3±108.0 (3)	0.61
Erythrocytes (10^6/µl)	12.3±0.7 (10)	13.2±1.2 (3)	0.55
Hemoglobin (g/dl)	19.0±0.8 (10)	20.4±1.7 (3)	0.53
Hematocrit (%)	63.3±1.4 (10)	66.7±2.6 (3)	0.32
**Clinical chemistry values**			
Urea (mg/dl)	49.4#	47.5#	n.a.
Creatinine (mg/dl)	0.27#	0.21#	n.a.
Cholesterol (mg/dl)	1330.0±140.1 (9)	1329.0±270.0 (4)	0.99
Triglycerides (mg/dl)	737.1±100.3 (9)	715.0±135.3 (4)	0.90

Values are mean±SEM (n). T-tests with Welch's correction. #pooled from n=4-9/group.

Suppl. table 3: Ldlr$^{-/-}$ mice after reconstitution with wildtype or Ccr2$^{-/-}$ bone marrow and ten weeks of a high fat diet

	**Wildtype**	***Ccr2$^{-/-}$***	**p-value**
**Clinical characteristics**			
Body weight (g)	26.0±1.6 (8)	23.5±1.2 (9)	0.53
Spleen weight (mg)	148.1±6.2 (8)	138.5±7.7 (9)	0.35
Rel. spleen weight (%)	0.6±0.03 (8)	0.6±0.04 (9)	0.72
Right kidney weight (mg)	192.6±11.5 (8)	181.7±6.4 (9)	0.44
Rel. right kidney weight (%)	0.7±0.05 (8)	0.8±0.03 (9)	0.80
**Hematological values**			
Total leukocytes (10^3/µl)	12.6±0.9 (8)	10.7±1.2 (9)	0.23
Monocytes (10^3/µl)	0.6±0.04 (8)	0.3±0.03 (9)	0.0002***
Lymphocytes (10^3/µl)	8.9±0.8 (8)	7.8±0.9 (9)	0.34
Granulocytes (10^3/µl)	3.1±0.2 (8)	2.7±0.4 (9)	0.36
Thrombocytes (10^3/µl)	1012.0±111.7 (8)	922.1±180.2 (9)	0.67
Erythrocytes (10^6/µl)	10.1±0.5 (8)	10.5±0.6 (9)	0.63
Hemoglobin (g/dl)	16.1±0.8 (8)	16.4±0.8 (9)	0.74
Hematocrit (%)	55.4±3.1 (8)	56.4±2.2 (9)	0.80
**Clinical chemistry values**			
Urea (mg/dl)	53.7±5.4 (2)#	61.5±4.1 (2)#	0.38
Creatinine (mg/dl)	0.67±0.05 (2)#	0.57±0.06 (2)#	0.36
Cholesterol (mg/dl)	1642±89.6 (7)	1358±161.9 (8)	0.15
Triglycerides (mg/dl)	896.6±91.9 (7)	738.8±58.7 (8)	0.18
**ELISA**			
CCL2 (pg/ml)	18.8±2.9 (5)	96.4±25.0 (7)	0.048*

Values are mean±SEM (n). T-test with Welch's correction. #pooled from n=8-9/group.

Suppl. table 4: Parallel gene regulation in kidney and aorta four weeks after pyelonephritis

	**Kidney**			**Aorta**		
	**Log2FC**	**p-value**	**adj. p-value**	**Log2FC**	**p-value**	**adj. p-value**
**Upregulated genes**						
Adgrg3	3.01	0.02	0.59	1.351	0.02	0.35
Akr1b8	1.30	0.03	0.73	1.885	0.00	0.08
Apol11b	1.93	0.01	0.55	4.573	0.00	0.10
Apol7c	4.64	0.02	0.68	3.472	0.01	0.25
Arid5a	1.28	0.00	0.20	1.299	0.03	0.42
Camp	2.85	0.00	0.39	4.389	0.01	0.18
Ccl8	2.50	0.00	0.03	4.193	0.00	0.00
Ccl9	1.20	0.00	0.05	1.508	0.00	0.16
Ccr2	1.17	0.00	0.11	1.329	0.00	0.05
Ccr5	1.04	0.02	0.58	1.366	0.01	0.19
Cd300a	1.39	0.00	0.11	1.628	0.01	0.29
Cd72	1.40	0.02	0.58	1.882	0.00	0.00
Clec4b1	3.18	0.01	0.41	1.436	0.01	0.23
Clec4n	2.35	0.00	0.35	5.029	0.00	0.00
Csf2rb	1.04	0.02	0.63	2.062	0.00	0.01
Cxcr2	3.09	0.02	0.58	2.663	0.01	0.17
Dpep2	2.14	0.00	0.36	2.820	0.02	0.40
ENSMUSG00000128977	1.12	0.00	0.23	1.382	0.00	0.01
ENSMUSG00000135102	1.22	0.00	0.05	2.085	0.00	0.00
Fcgr4	1.50	0.04	0.78	1.727	0.00	0.05
Gm42600	4.00	0.05	0.78	2.549	0.02	0.40
Gm43359	3.02	0.04	0.77	1.794	0.04	0.48
Hspa1b	1.18	0.00	0.06	1.141	0.02	0.38
Ifitm6	1.52	0.01	0.55	2.074	0.02	0.36
Ighg1	1.64	0.05	0.78	2.144	0.00	0.02
Iigp1c	1.05	0.03	0.73	1.322	0.00	0.06
Kcnk10	3.64	0.02	0.59	4.278	0.01	0.22
Klra2	4.08	0.00	0.35	1.529	0.04	0.47
Lrg1	1.78	0.00	0.03	1.454	0.00	0.00
Ly6c2	1.30	0.01	0.48	2.078	0.00	0.13
Ly6g	4.64	0.00	0.35	4.132	0.04	0.48
Mefv	4.01	0.04	0.75	4.448	0.02	0.34
Mmp8	1.72	0.03	0.68	2.135	0.01	0.23
Mmp9	1.67	0.00	0.25	2.618	0.00	0.00
Mpeg1	1.05	0.00	0.37	1.742	0.00	0.00
Ms4a7	1.30	0.00	0.34	1.788	0.00	0.00
Ncf2	1.06	0.01	0.44	1.132	0.03	0.46
Pglyrp1	2.25	0.00	0.38	3.467	0.00	0.02
Pirb	1.19	0.00	0.35	2.355	0.00	0.01
Retnlg	2.06	0.01	0.52	3.030	0.00	0.01
S100a8	1.64	0.03	0.71	2.894	0.00	0.00
Selp	1.40	0.00	0.03	3.057	0.02	0.33
Serpine1	1.03	0.00	0.16	2.292	0.04	0.49
Slfn4	1.98	0.01	0.53	3.154	0.00	0.11
Trim30a	1.00	0.01	0.55	1.293	0.00	0.00
Wfdc21	1.22	0.03	0.69	4.480	0.00	0.10
Zbp1	1.04	0.03	0.70	2.574	0.00	0.00
Adgrg3	3.01	0.02	0.59	1.351	0.02	0.35
**Downregulated genes**						
Or8b40	-2.76	0.00	0.09	-3.06	0.03	0.43
Gm56503	-1.52	0.00	0.38	-1.66	0.03	0.42
Pln	-1.09	0.02	0.58	-1.63	0.02	0.34
Gm10603	-4.67	0.03	0.74	-3.98	0.01	0.31
Zfp128	-4.29	0.04	0.76	-2.92	0.04	0.50
ENSMUSG00000123750	-2.24	0.04	0.78	-3.44	0.02	0.33

Supplemental figures:

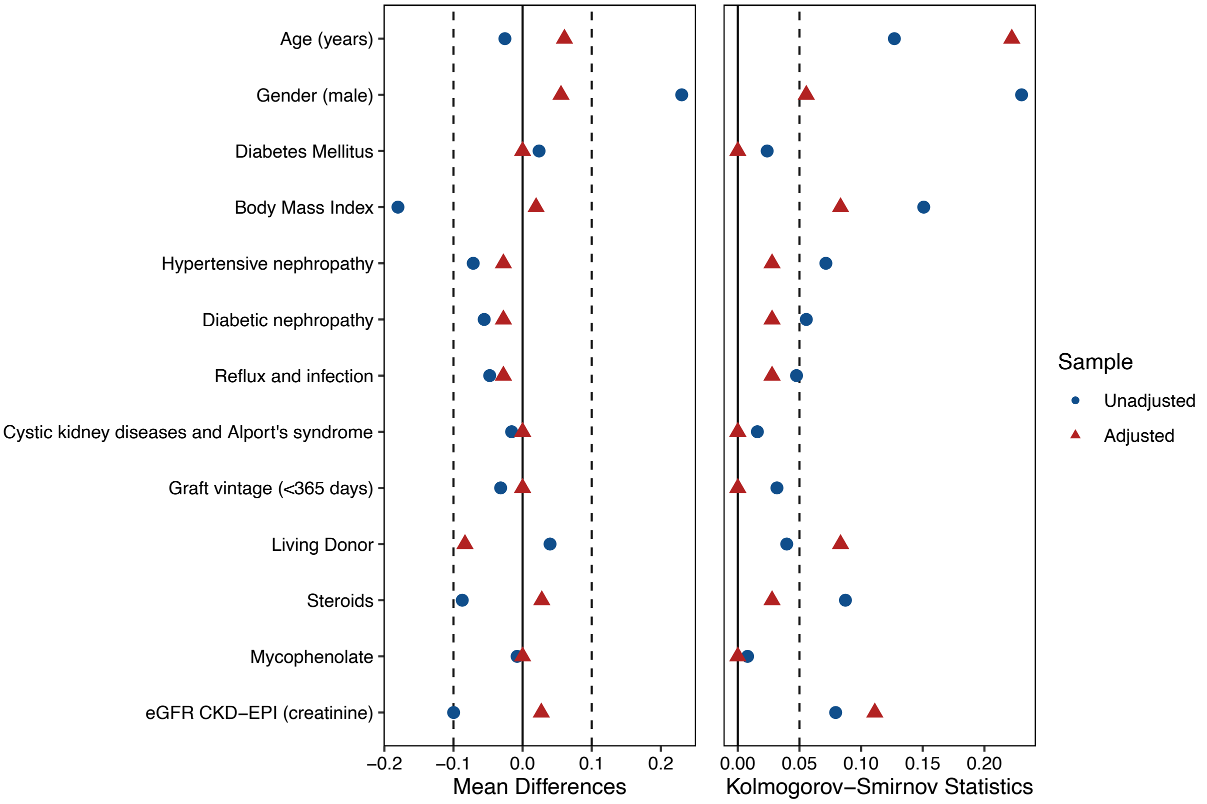

Supplemental figure 1: Covariate balance after propensity score matching

Standardized mean differences (SMD) and Kolmogorov-Smirnov test results for each variable before (blue circles) and after (red triangles) propensity score matching (PSM) are shown.

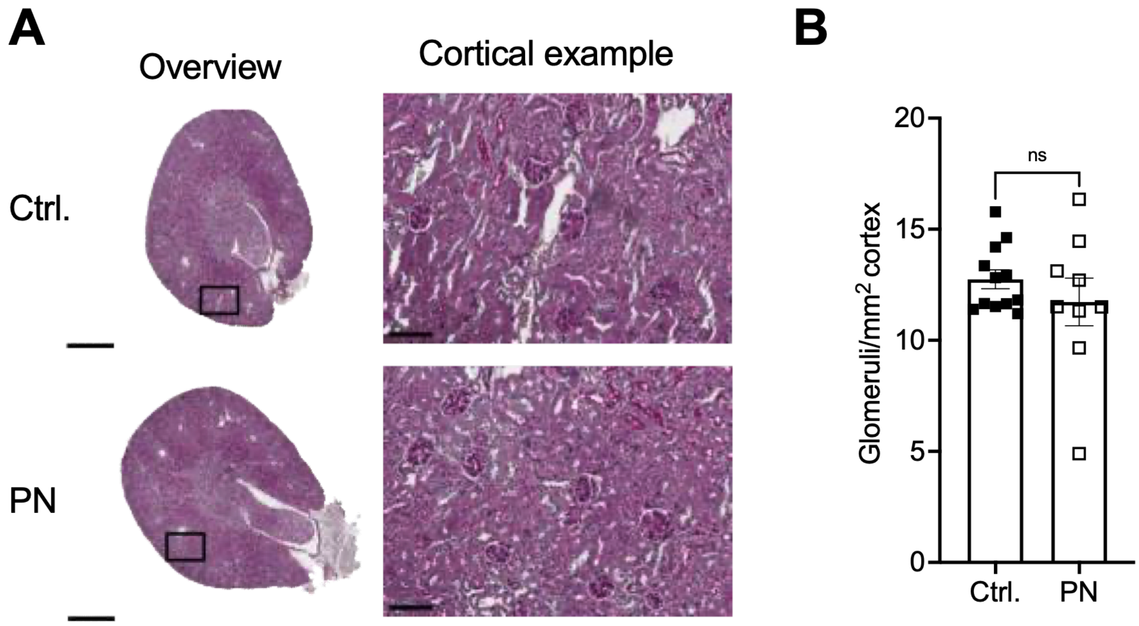

Suppl. figure 2: Kidney histology in Ldlr$^{-/-}$ mice 11 weeks after pyelonephritis

(A.B) Atherosclerosis was promoted by high fat diet for ten weeks starting one week after induction of pyelonephritis (PN) in female *Ldlr*$^{-/-}$ mice. Representative PAS-stained sections are shown (size bars indicate 1mm in overview and 100µm in cortical examples. B: quantification in n=9-12 mice in 7 indep. exp.. t–tests with Welch's correction).

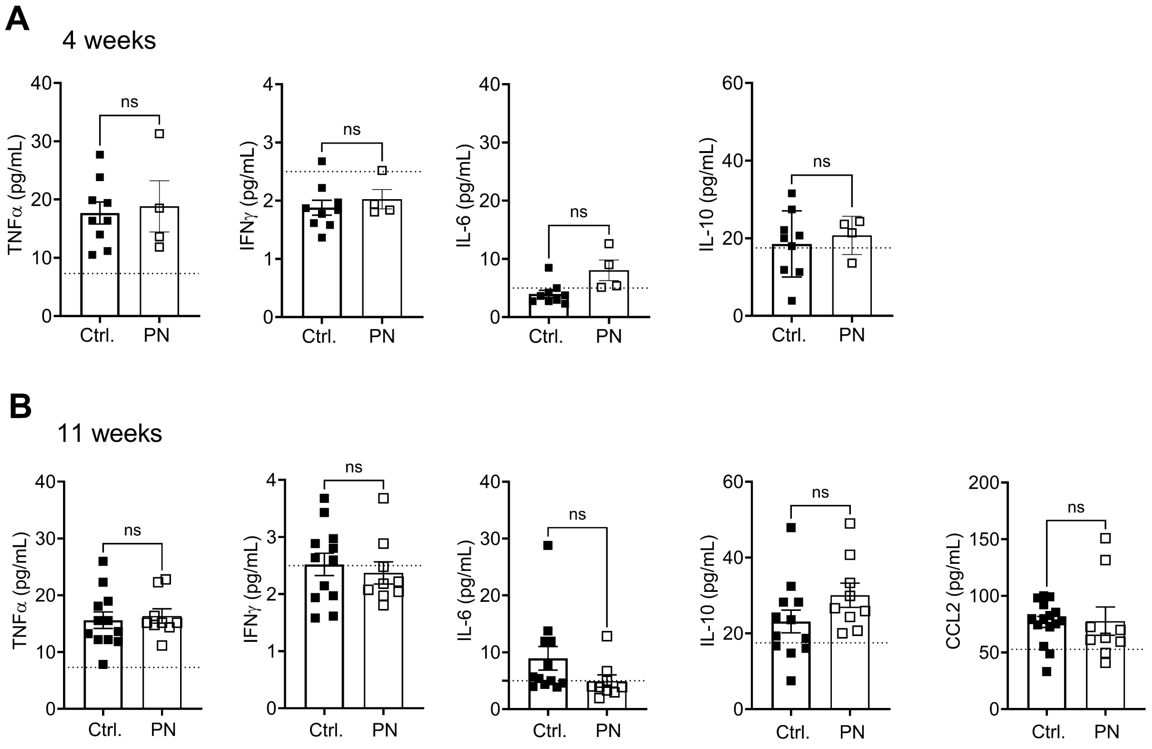

Suppl. figure 3: Systemic cytokine levels during atherosclerosis induction after pyelonephritis

(A.B) In *Ldlr*$^{-/-}$ mice treated as described in figure 1A. serum TNFα. IFNγ. IL-6. IL-10 and CCL2 were analyzed with a cytometric bead assay four (A) and eleven weeks after kidney infection (PN) (A: n=4-9. 3 indep. exp.. B n=9-12. 7 indep. exp. t–tests with Welch's correction. dotted lines indicate detection limit).

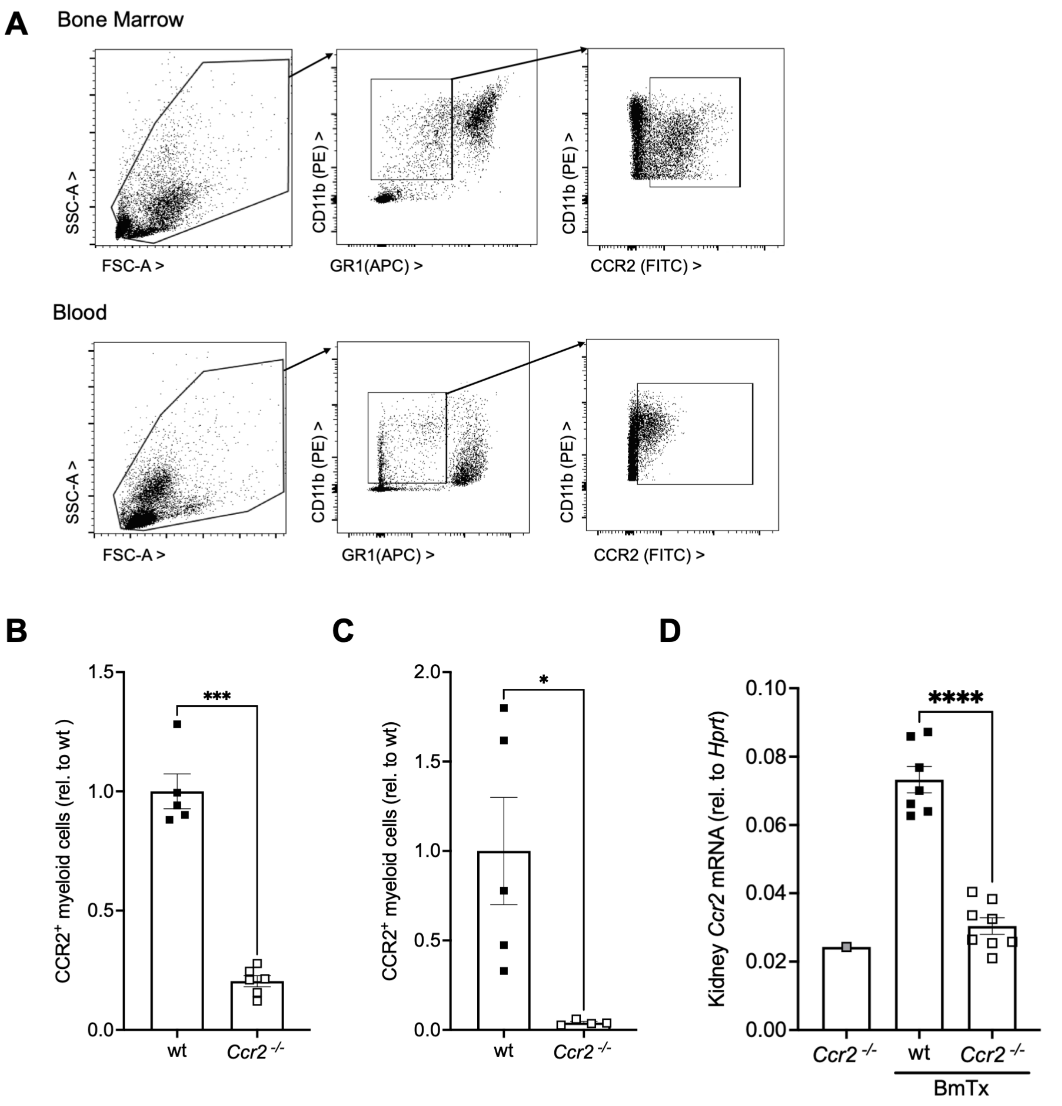

Suppl. figure 4: CCR2 assessment in complete bone marrow chimeric Ldlr$^{-/-}$ mice

(A-D) *Ldlr$^{-/-}$* mice reconstituted with *Ccr2$^{-/-}$* or control wildtype bone marrow were sacrificed after ten weeks of a high fat diet starting one week after induction of pyelonephritis (as depicted in figure 2B. **(A-C)** Assessment of bone marrow (B) and blood (C) myeloid CCR2 surface expression (A. gating strategy. B.C statistical analysis of n=4-6. 3 indep. transplantations. t–tests with Welch's correction). **(D)** Renal *Ccr2* mRNA expression was assessed by qPCR (n=7-8 from 4 indep. transplantations. t–test with Welch's correction. a *Ccr2$^{-/-}$* mouse serving as negative control).

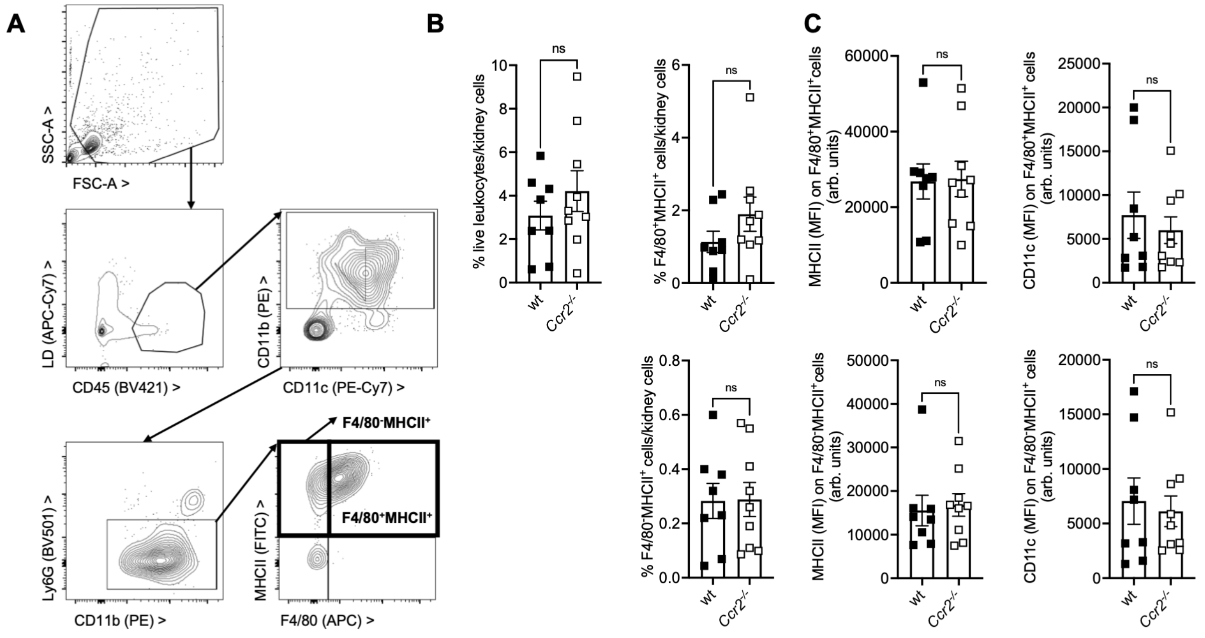

Suppl. figure 5: Kidney macrophage characterization after PN in the absence and presence of Ccr2

(A-C) *Ldlr*$^{-/-}$ mice were lethally irradiated and reconstituted with *Ccr2*$^{-/-}$ or control wildtype bone marrow (BM) and sacrificed after ten weeks of a high fat diet starting one week after induction of pyelonephritis (PN) as outlined in figure 2B. Renal leukocyte enumeration by flow cytometry (A. gating strategy and B. statistical analysis frequencies (B) and mean surface expression levels (C) of n=8-9/group in 5 indep. exp.. t-tests with Welch's correction).

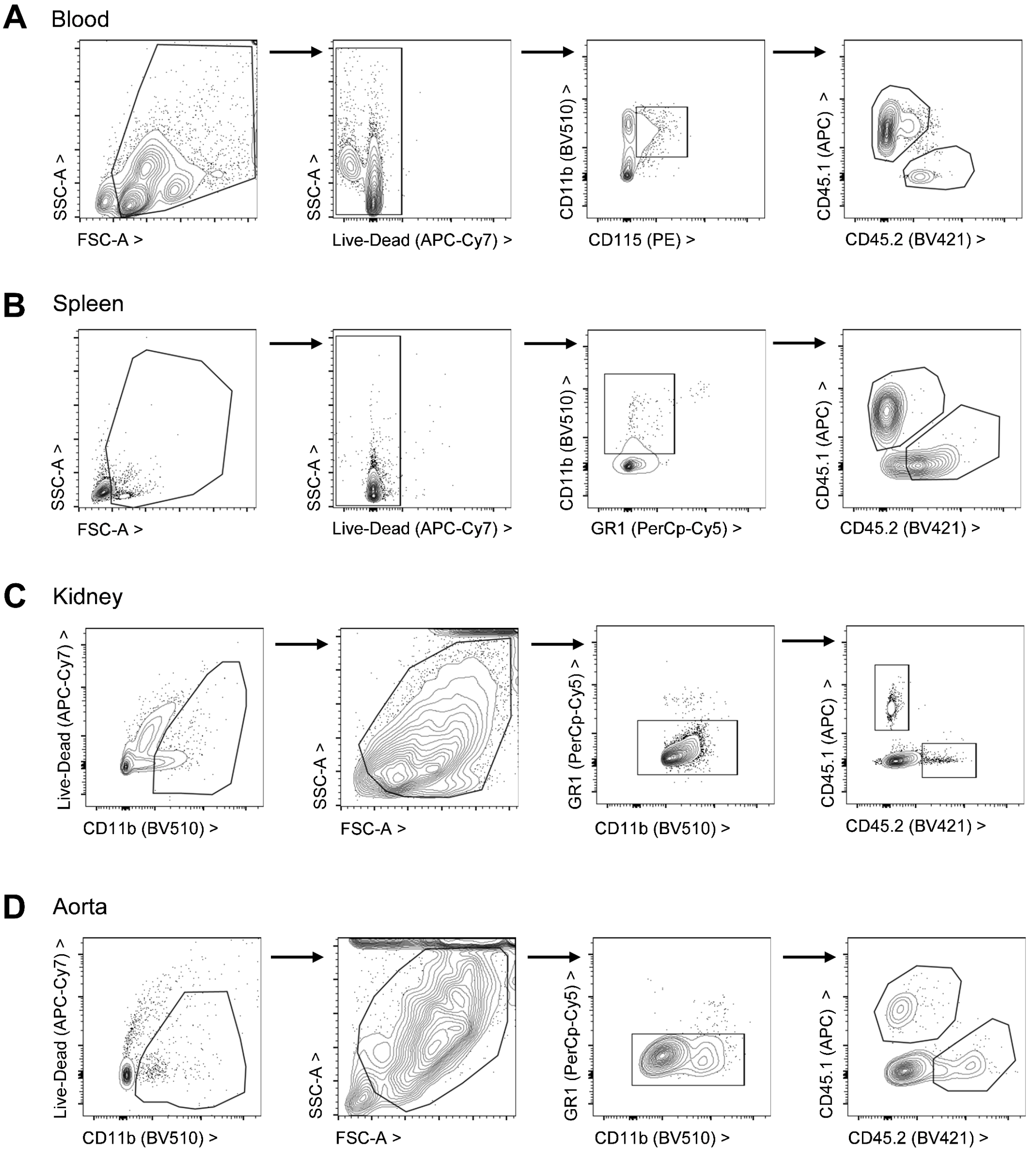

Suppl. figure 6: Flow cytometric gating strategy for the assessment of mixed bone marrow chimeras.

(A-D) Gating strategies for blood monocytes (A) and spleen (B). kidney (C). and aortic (D) leukocytes for $CD11b^+$ myeloid cells and CD45.1 and CD45.2 congenic markers are shown.

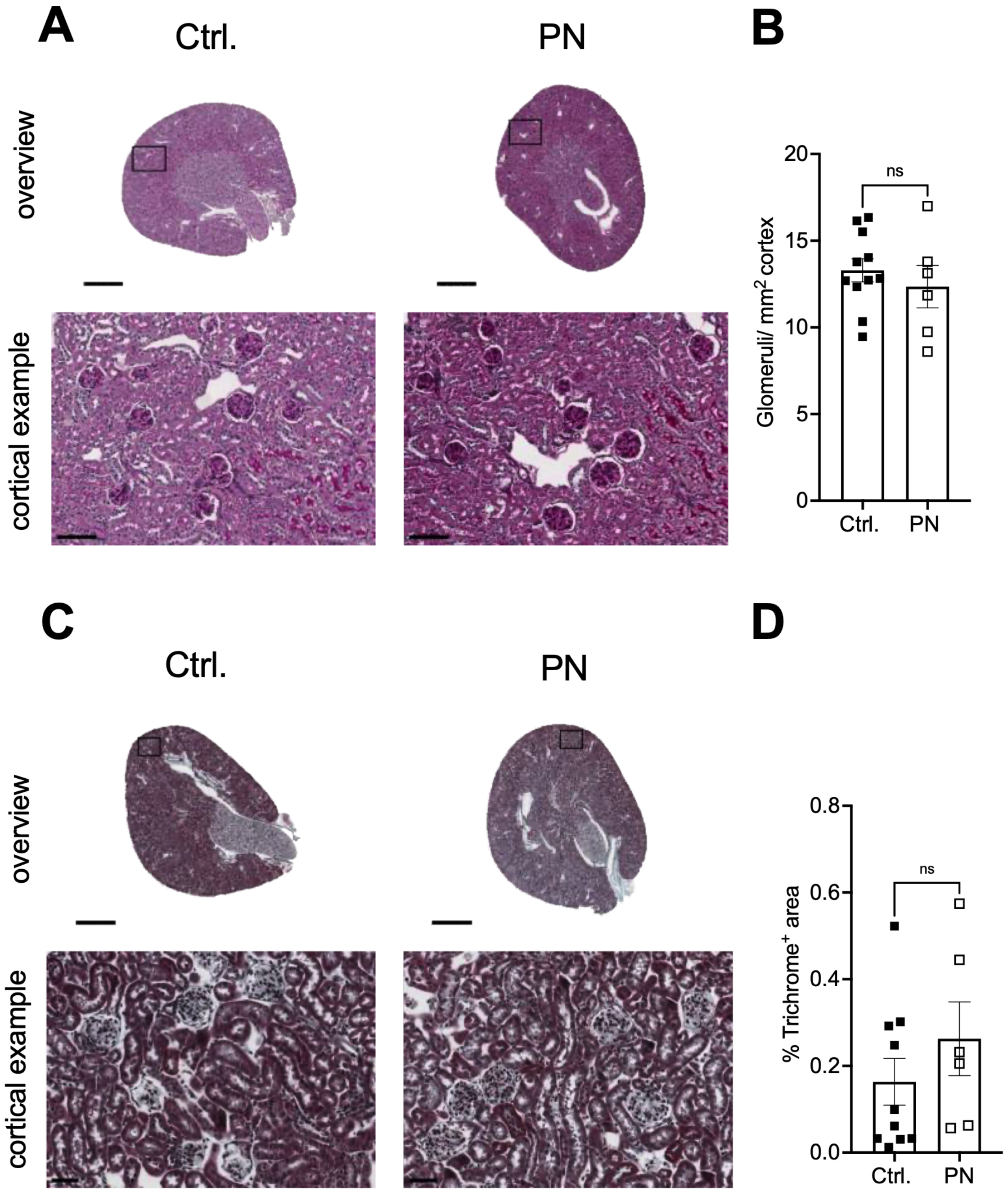

Suppl. figure 7: Kidney histology in Ldlr$^{-/-}$ mice four weeks after pyelonephritis

(A-D) Atherosclerosis was promoted by high fat diet for three weeks starting one week after induction of pyelonephritis (PN) in female *Ldlr*$^{-/-}$ mice and renal histology assessed after PAS (A. B) and trichrome staining for fibrosis assessment (C.D) (A. C examples. size bars indicate 1mm and 100µm and B.C. quantification in n=6-11 mice in 4 indep. exp.. t–tests with Welch's correction).

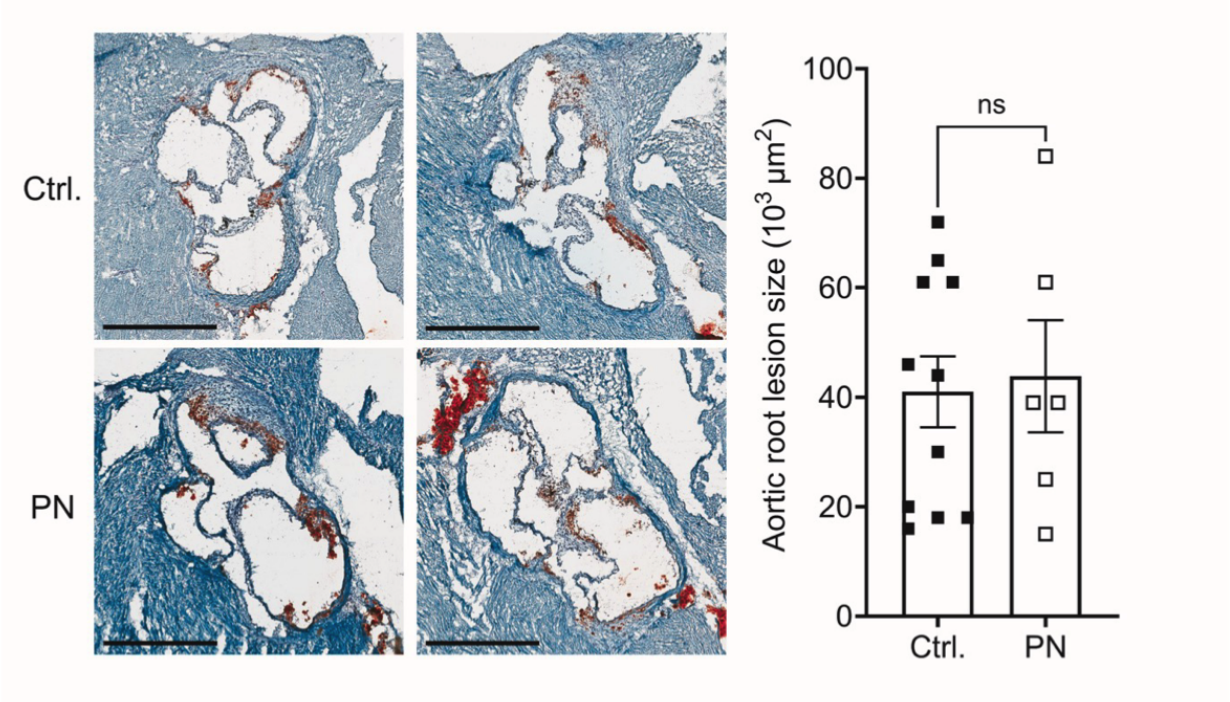

Suppl. figure 8: Aortic lesion size in Ldlr$^{-/-}$ mice after three weeks of a high fat diet and four weeks after pyelonephritis

Atherosclerosis was promoted by high fat diet for ten weeks starting one week after induction of pyelonephritis (PN) in female *Ldlr*$^{-/-}$ mice. Examples of Oil-red-O-stained aortic roots and results of statistical analysis are shown (size bars indicate 500µm. n=6-11 mice in 4 indep. exp. t–test with Welch's correction).

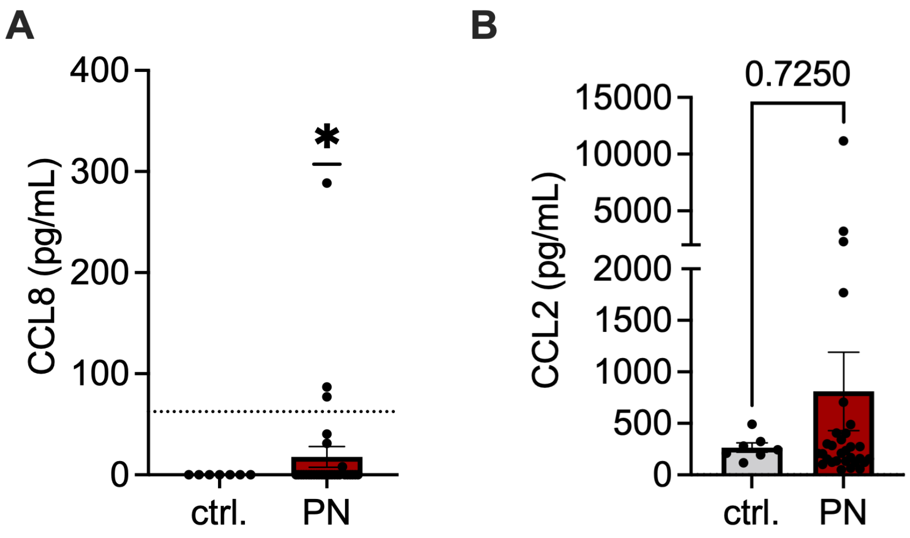

Figure 9: Systemic CCL2 and CCL8 regulation human pyelonephritis

(A,B) Plasma from patients with acute pyelonephritis (PN) was assessed by ELISA for CCL2 (D) and CCL8 (E. n=7 ctrl. and 30 PN. one-sided test of CCL8 detection. CCL2: t–test with Welch's correction).

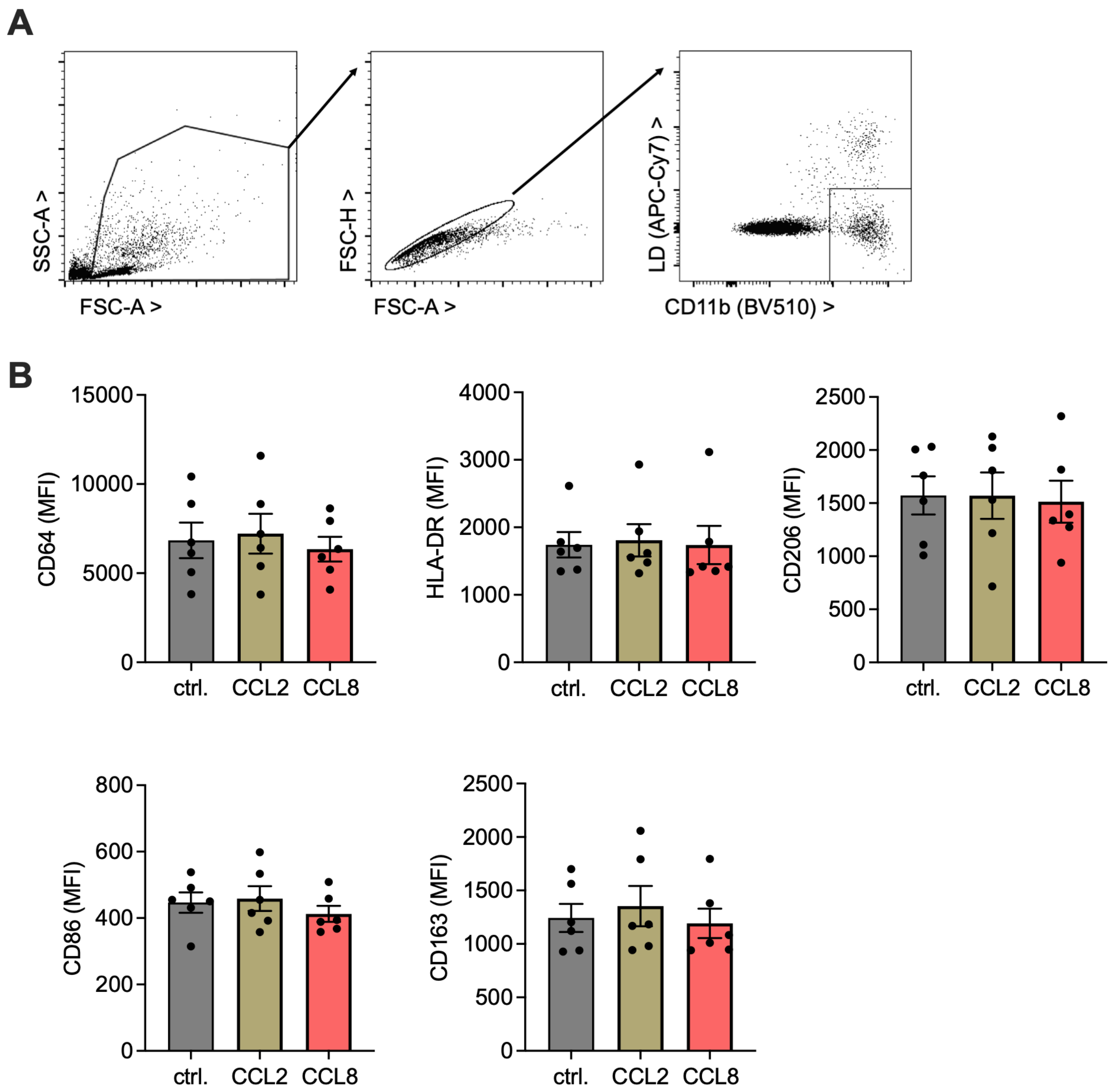

Figure 10: Impact of CCL2 and CCL8 on human monocyte-derived macrophage differentiation

(A,B) Human primary monocyte-derived macrophages were stimulated with 100ng/ml CCL2 or CCL8 during 7 days differentiation. Surface expression of M1 type markers CD64, HLA-DR APC type marker CD86, M2 type marker CD206 and CD163 U(hemoglobin scavenger) was assessed by flow cytometry (Gating in A, B: Mean fluorescence intensities (MFI) or the indicated markers from n=6 donors in 3 indep. exp., ANOVAs not significant).
